# Anti-inflammatory signaling by mammary tumor cells mediates prometastatic macrophage polarization in an innovative intraductal mouse model for triple-negative breast cancer

**DOI:** 10.1186/s13046-018-0860-x

**Published:** 2018-08-15

**Authors:** Jonas Steenbrugge, Koen Breyne, Kristel Demeyere, Olivier De Wever, Niek N. Sanders, Wim Van Den Broeck, Cecile Colpaert, Peter Vermeulen, Steven Van Laere, Evelyne Meyer

**Affiliations:** 1 Laboratory of Biochemistry, Department of Pharmacology, Toxicology and Biochemistry, Faculty of Veterinary Medicine, Ghent University, Merelbeke, Belgium; 2 Translational Cancer Research Unit Antwerp, Center for Oncological Research, General Hospital Sint-Augustinus, Wilrijk, Belgium; 3 Cancer Research Institute Ghent (CRIG), Ghent, Belgium; 4 Laboratory of Experimental Cancer Research, Department of Radiation Oncology and Experimental Cancer Research, Ghent University, Ghent, Belgium; 5 Laboratory of Gene Therapy, Department of Nutrition, Genetics and Ethology, Faculty of Veterinary Medicine, Ghent University, Merelbeke, Belgium; 6 Department of Morphology, Faculty of Veterinary Medicine, Ghent University, Merelbeke, Belgium; 7 Department of Pathology, General Hospital Sint-Augustinus, Wilrijk, Belgium; 8 Present address: Department of Pharmacological and Physiological Sciences, The University of Chicago, Chicago, IL USA

**Keywords:** Triple-negative breast cancer, Intraductal model, 4T1 mammary tumor cells, RAW264.7 macrophages, Chitinase 3-like 1, Lipocalin 2

## Abstract

**Background:**

Murine breast cancer models relying on intraductal tumor cell inoculations are attractive because they allow the study of breast cancer from early ductal carcinoma in situ to metastasis. Using a fully immunocompetent 4T1-based intraductal model for triple-negative breast cancer (TNBC) we aimed to investigate the immunological responses that guide such intraductal tumor progression, focusing on the prominent role of macrophages.

**Methods:**

Intraductal inoculations were performed in lactating female mice with luciferase-expressing 4T1 mammary tumor cells either with or without additional RAW264.7 macrophages, mimicking basal versus increased macrophage-tumor cell interactions in the ductal environment. Imaging of 4T1-derived luminescence was used to monitor primary tumor growth and metastases. Tumor proliferation, hypoxia, disruption of the ductal architecture and tumor immune populations were determined immunohistochemically. M1- (pro-inflammatory) and M2-related (anti-inflammatory) cytokine levels were determined by Luminex assays and ELISA to investigate the activation state of the macrophage inoculum. Levels of the metastatic proteins matrix metalloproteinase 9 (MMP-9) and vascular endothelial growth factor (VEGF) as well as of the immune-related disease biomarkers chitinase 3-like 1 (CHI3L1) and lipocalin 2 (LCN2) were measured by ELISA to evaluate disease progression at the protein level.

**Results:**

Mice intraductally co-injected with macrophages showed severe splenomegaly with faster ductal breakthrough of tumor cells and increased metastases in axillary lymph nodes and lungs. These mice showed higher M1-related cytokines in the early disease stages (at 1 to 3 weeks post-inoculation) due to the pro-inflammatory nature of RAW264.7 macrophages with increased Ly6G-positive neutrophils and decreased anti-inflammatory macrophages in the tumor microenvironment. However, upon metastasis (at 5 weeks post-inoculation), a prominent increase in M2-related cytokine levels was detected and established a tumor microenvironment with similar immune populations and cytokine responses as in mice which received only 4T1 tumor cells. The observed tumor-associated immune responses and the increased metastasis were associated with significantly induced local and systemic levels of MMP-9, VEGF, CHI3L1 and LCN2.

**Conclusions:**

The current experimental study with an innovative immunocompetent intraductal model for TNBC pinpoints towards a metastasis-supporting M1 to M2 macrophage polarization in the mammary ducts mediated by 4T1-derived signaling. We propose to explore this process as immunotherapeutic target.

**Electronic supplementary material:**

The online version of this article (10.1186/s13046-018-0860-x) contains supplementary material, which is available to authorized users.

## Background

Breast cancer is one of the most prevalent cancers in women and especially the aggressive subtypes, including triple-negative breast cancer (TNBC), remain difficult to treat with traditional chemotherapeutics directly targeting the tumor cells [1]. As an alternative treatment, novel immunotherapeutic strategies now focus on targeting the stromal components in the breast tumor microenvironment [2, 3]. However, testing of these promising therapies as well as identification of druggable immune targets relies on preclinical mouse models that faithfully mimic breast tumor immunology and tumor progression [4, 5]. We recently introduced an innovative mouse model for TNBC that relies on the inoculation of syngeneic 4T1 murine mammary tumor cells through the teat canal directly into the mammary ducts (i.e. the luminal mammary gland compartment) of immunocompetent and lactating BALB/c mice, recapitulating the early ductal carcinoma in situ (DCIS) stage at its onset. Upon comparison to classical fat pad tumors, intraductal tumors showed a slower development, yet metastasized at a similar rate in the presence of potent tumor-associated immune responses [6].

In the current study, we aimed to investigate the immune responses in this innovative intraductal screening model mediated by macrophage-tumor cell interactions. Macrophages are the most common innate immune cells in the breast tumor microenvironment and are now recognized to act as regulators in multiple breast cancer-associated processes based on their phenotype (either M1 or M2) [7–11]. Tumor-associated macrophages (TAM) are characterized as a M2 macrophage population producing key mediators such as matrix metalloproteinases (including MMP-9) and vascular endothelial growth factor (VEGF) that stimulate breakdown of the extracellular matrix (promoting tumor cell invasion) and angiogenesis (promoting tumor cell survival and metastasis), respectively [7, 8]. Moreover, in contrast to M1 macrophages that have antitumoral capacities, M2 macrophages lack tumoricidal (pro-inflammatory) activity and exert anti-inflammatory immune responses which prevent the host of mounting an effective counterattack against the tumor and thereby stimulate tumor growth and metastasis [7, 8]. By intraductally inoculating 4T1 mammary tumor cells with or without RAW264.7 macrophages, we verified that the presence of these latter macrophages in the ductal environment stimulates 4T1 ductal breakthrough, metastasis and splenomegaly. Investigating the education of macrophages by mammary tumor cells, we showed that the increased metastasis in 4T1 and RAW264.7 co-inoculated mice is associated with a shift from high pro- to anti-inflammatory cytokine levels, indicative for a M1 to M2 macrophage polarization. This prometastatic event is also corroborated by local and systemic MMP-9 and VEGF levels. Furthermore, local and systemic levels of CHI3L1 and LCN2, two immune-related biomarkers used for prognosticating and monitoring disease in breast cancer patients and mice [6, 12–14], provided an additional verification of the tumor progression in 4T1 inoculated mice with and without RAW264.7 macrophages.

Taken together, this study investigated the effects of additional macrophages and their polarization on ductal tumor cell breakthrough and metastasis using a 4T1-based intraductal model to define novel avenues for the clinical translation of immunomodulatory applications against TNBC.

## Methods

### Animals

Female BALB/c mice were bred, housed and fed *ad libitum* in a controlled facility with a light/dark cycle of 12 h. All research involving animals was performed in accordance with the recommendations in the Guide for the Care and Use of Laboratory Animals of the National Institutes of Health. The study protocols were approved by the Committee on the Ethics of Animal Experiments of The Faculty of Veterinary Medicine at Ghent University (approval number: EC2015/127).

### 4T1 and RAW264.7 cell culture

The BALB/c-derived 4T1 mammary tumor cell line used in this study constitutively expresses the firefly luciferase gene and was a kind gift from Prof. Clare Isacke (Breakthrough Breast Cancer Research Centre, London, UK). This tumor cell line resembles the aggressive phenotype and metastasis seen in human TNBC (estrogen receptor (ER)-negative, progesterone receptor (PR)-negative and human epidermal growth factor receptor 2 (HER2)-negative) [15, 16]. The BALB/c-derived RAW264.7 macrophage cell line was a kind gift from Prof. Rudi Beyaert (Unit of Molecular Signal Transduction in Inflammation, Inflammation Research Center, Ghent University-VIB, Ghent, Belgium). Both cell lines were maintained in Dulbecco’s Modified Eagle’s Medium (DMEM) supplemented with 10% heat-inactivated fetal bovine serum (FBS), 100 U/ml penicillin and 100 μg/ml streptomycin (Thermo Fisher Scientific, Waltham, MA, USA) in culture flasks. Harvesting of cultured 4T1 cells was performed using 0.25% trypsin- ethylenediaminetetraacetic acid (EDTA) (Thermo Fisher Scientific), whereas RAW264.7 macrophages were harvested using a cell scraper. The harvested cells were subsequently washed through centrifugation (805 g for 5 min) and the cell pellets were resuspended in phosphate buffered saline (PBS). Cell numbers were determined through counting using a Bürker chamber.

For preliminary in vitro experiments, 4T1 mammary tumor cells and RAW264.7 macrophages were cultured either alone (5 × 10^5^ cells in mono-culture) or together (5 × 10^5^ of each cell type in co-culture) supplemented with 1 ml of cell culture medium per well in 24 well plates. The cell cultures were incubated (37 °C, 5% CO_2_) for 24 h (to examine CHI3L1 and LCN2 secretion) or 96 h (to examine RAW264.7 macrophage polarization) with daily change of the cell culture medium. The harvested cell culture media were spun down (17,000 g) for 10 min to remove cellular debris for further analyses. Cells from 3 wells of 96 h RAW264.7 mono- and 4T1 + RAW264.7 co-cultures were harvested using a cell scraper, pooled and washed through centrifugation (805 g for 5 min) for subsequent flow cytometric analysis.

### Flow cytometric analysis of RAW264.7 macrophage polarization

Harvested and pelleted cells from RAW264.7 mono- and 4T1 + RAW264.7 co-cultures were suspended in 2.5 ml FACS buffer (containing PBS, 1% bovine serum albumin (BSA), 2.5 mM EDTA and 0.01% sodium azide) and 100 μl of the cell suspension was plated in a well of a 96 well plate for counting through flow cytometry (Analis, Cytoflex). Propidium iodide (PI, 2 μl at 50 μg/ml) was also added to the well to evaluate the viability of the cells. Remaining cell suspensions were plated at 100 μl per well in a 96 well plate and the well plate was centrifuged to pellet the cells (805 g for 5 min). To block Fc receptors found on the RAW264.7 macrophages, cell pellets were subsequently resuspended in FcR blocking reagent (1:10 diluted in FACS buffer; Miltenyi Biotec, Leiden, Netherlands) and incubated for 10 min at 2–8 °C. Following centrifugation, cell pellets derived from 4T1 + RAW264.7 co-cultures were stained for 30 min at 2–8 °C with APC-labeled anti-F4/80 (diluted 1:20 in FACS buffer; clone CI:A3–1; Bio-Rad, CA, USA) to distinguish RAW264.7 macrophages from 4T1 tumor cells. This staining was not performed on cells derived from RAW264.7 mono-cultures as no distinction is needed between RAW264.7 macrophages and 4T1 tumor cells. To allow intracellular staining, the pelleted cells were fixed using BD Cytofix/Cytoperm solution (Becton Dickinson, Erembodegem, Belgium) for 20 min at 2–8 °C and permeabilized afterwards by washing twice in 1× BD Perm/Wash Buffer (Becton Dickinson). Cell pellets derived from RAW264.7 mono- and 4T1 + RAW264.7 co-cultures were stained for 30 min at 2–8 °C with PE-labeled anti-IL-12 (diluted 1:20 in 1× BD Perm/Wash Buffer; clone B211220; BioLegend, CA, USA) or anti-TGF-β1 (diluted 1:40 in 1× BD Perm/Wash Buffer; clone TW7-16B4; BioLegend). Istoype-matched and autofluorescence controls were also included for analyses. Following cellular stainings, cell pellets were washed twice with 1× BD Perm/Wash Buffer prior to analysis with a flow cytometer. A maximum of 5% overlap with the isotype control signal was allowed when setting the gates to identify positive F4/80, IL-12 and TGF-β1 signals.

### Intraductal inoculations with 4T1 and/or RAW264.7 cells

Intraductal inoculations were conducted in the third mammary gland pair of female lactating BALB/c mice under inhalation anesthesia (induction: 2–3% isolflurane supplemented with oxygen (O_2_); maintenance: 1–1.5% isoflurane supplemented with O_2_) in combination with intraperitoneal (i.p.) administration of buprenorphine (long-acting analgesic, 10 μg/kg Vetergesic, Patheon UK Ltd., Swindon, UK). To attain full lactation, female BALB/c mice of 8–13 weeks (w) were mated with male BALB/c mice of 10 w and pups were weaned 12–14 days following delivery. Mice were inoculated through the mammary teat canal 1 h after weaning using a 32-gauge blunt needle with either 5 × 10^4^ 4T1 cells, 5 × 10^4^ RAW264.7 macrophages or 5 × 10^4^ of both 4T1 cells and RAW264.7 macrophages, suspended in a 100 μl mixture of 1:10 PBS and Matrigel^®^ (Corning, Bedford, MA, USA). All materials used for inoculation were kept cold to prevent clotting of the Matrigel^®^.

### Analysis of primary tumor and metastasis progression

Intraductally inoculated mice were screened weekly through in vivo bioluminescence imaging using the IVIS lumina II system (PerkinElmer, Zaventem, Belgium) to monitor growth of 4T1 firefly luciferase-expressing primary tumors. Mice were injected i.p. with 200 μl D-luciferin suspended in PBS (2 mg/100 μl; Gold Biotechnology, St. Louis, MO) and approximately 10 min later images were acquired under inhalation anesthesia with isoflurane. Metastases were detected through ex vivo bioluminescence imaging, which required euthanasia of the intraductally inoculated mice and subsequent harvesting of metastases-bearing organs. Mice were therefore sedated by i.p. injection of a mixture of 100 mg/kg ketamine (Ketamidor, Ecuphar nv/sa, Oostkamp, Belgium) and 10 mg/kg xylazine (Xylazini Hydrochloridum, Val d’Hony-Verdifarm, Beringen, Belgium) and then sacrificed through cervical dislocation. Axillary lymph nodes and lungs were isolated and screened for 4T1 metastases. Primary tumors and spleens were also isolated and weighed upon sacrification. Quantification of the 4T1-derived bioluminescent signals calculated by the living image analysis software 3.2 from in vivo and ex vivo images was performed by dividing the measured total flux with the selected area.

### Histology and immunohistochemistry

Primary tumors, mammary glands, and metastases-bearing axillary lymph nodes and lungs were isolated upon sacrifice of the mice, fixed in buffered 3.5% formaldehyde for 24 h at room temperature (RT) and embedded in paraffin wax. Sections (5 μm) were deparaffinized, hydrated and stained with hematoxylin and eosin (H&E). Sections were then dehydrated and mounted with a cover glass for evaluation.

For immunohistochemical stainings, sections of 2–3 μm were deparaffinized and antigen retrieval was performed with citrate buffer (10 mM tri-sodium citrate (Santa Cruz Biotechnology, Heidelberg, Germany)), pH 6, (for Ki67, carbonic anhydrase IX (CAIX), CD45, Ly6G, CD163, CD8a, CD31, F4/80) or with Tris-EDTA buffer (10 mM Tris, 1 mM EDTA (Thermo Fisher Scientific)), pH 9, (for cytokeratin 5) both supplemented with 0.05% Tween-20 (Sigma-Aldrich, Bornem, Belgium) at 95 °C under pressure for 30 min using a Decloaking Chamber NxGen (Biocare Medical, CA, USA). The slides were allowed to cool down to RT for 30 min, after which endogenous peroxidase activity was blocked with 3% H_2_O_2_ in methanol (for Ki67, CAIX, cytokeratin 5, CD45, Ly6G, CD163, CD31 and F4/80) or 0.6% H_2_O_2_ in methanol (for CD8a) for 10 min at RT. Serum-free protein block (Dako, Heverlee, Belgium) was applied for 10 min at RT to block non-specific binding sites. The slides were subsequently incubated with primary antibody diluted in Antibody Diluent (Dako) for 1 h at RT and secondary antibodies were incubated for 30 min at RT. Primary antibodies and dilutions used were: anti-Ki67 (1:50; clone SP6; Thermo Fisher Scientific); anti-CAIX (1:1000; NB100–417; Novus Biologicals, Littleton, CO, USA); anti-cytokeratin 5 (1:100; clone EP1601Y; Abcam, Cambridge, UK); anti-CD45 (1:1000; clone 30-F11; Thermo Fisher Scientific); anti-Ly6G (1:1000; clone 1A8; BioLegend); anti-CD163 (1:500; clone EPR19518; Abcam); anti-CD31 (1:2000; clone EPR17259; Abcam); anti-F4/80 (1:10; clone CI:A3–1; Bio-Rad); anti-CD8a (1:50; clone 4SM15; Thermo Fisher Scientific). Secondary antibodies used were: Rat-on Mouse HRP-Polymer (Biocare Medical) for CD45, Ly6G, F4/80 and CD8a; Dako EnVision+ Rabbit (Dako) for Ki67, CAIX, cytokeratin 5, CD163 and CD31. A 3,3′-diaminobenzidine (DAB)-containing buffer (generated by adding 1 drop of Dako DAB+ chromogen to 1 ml of Dako DAB+ substrate buffer) was applied for 10 min at RT, which created a brownish signal that was visualized as a positive stain on the slide following counterstaining in hematoxylin for 3–4 min at RT. All rinsing steps in between the incubation steps were performed by applying tris-buffered saline (TBS, Biocare Medical) 3 times for 2 min at RT. All incubation and rinsing steps from peroxidase activity blocking to DAB chromogen staining were performed in a closed microscope slide box (humidified with TBS-wetted tissue paper) on an orbital shaker at 20 rpm. Next to qualitative assessment, differences in immunohistochemical staining between the inoculation groups at each time point were quantitated with ImageJ through color deconvolution (3 color split) and automatic counting.

### Cytokine profile analysis and measurement of protein levels

Primary tumors, mammary glands, spleens, axillary lymph nodes and lungs were isolated and homogenized. The homogenate was subsequently mixed with 300 μl lysis buffer supplemented with protease inhibitors (1% Nonidet P-40, 10 mM Tris-HCl at pH 7.4, 200 mM NaCl, 5 mM EDTA, 10% glycerol, 100 μM phenylmethylsulfonyl (PMSF), 1 mM oxidized L-glutathione (all from Sigma-Aldrich), 0.15 μM aprotinin and 2.1 μM leupeptin (Roche, Mannheim, Germany)). The suspensions were frozen overnight to allow their lysis and centrifuged twice at 17,000 g for 1 h at 4 °C the following day to precipitate the pellet. Protein concentration of the lysates was determined using the Bradford Protein Assay (BioRad, Hercules, CA) followed by spectrophotometrical measurement (Genesys 10S). Lysates were diluted to a concentration of 5 μg/μl with lysis buffer for further analysis. Serum was prepared from cardiac puncture harvested blood that was clotted through incubation at 37 °C for 1 h and then centrifuged at 17,000 g for 1 h at 4 °C.

A selected profile of cytokines (BAFF, G-CSF, IFN-γ, IL-1β, IL-4, IL-6, IL-10, MCP-1, MIP-2 and TNF-α) was quantified in lysates (50 μg protein), sera (diluted 1:4 in assay diluent) and culture media (undiluted) using Luminex Multiplex Assays (ProcartaPlex from Thermo Fisher Scientific) according to the manufacturer’s instructions. TGF-β1, MMP-9, VEGF, CHI3L1 and LCN2 levels were measured in lysates and sera using enzyme-linked immunosorbent assay (ELISA) according to the manufacturer’s instructions (TGF-β1: Mouse uncoated ELISA Kit, Thermo Fisher Scientific; MMP-9, VEGF, CHI3L1 and LCN2: Mouse Quantikine ELISA Kit, Biotechne, Minneapolis, MN, USA). A microplate reader was used to measure the absorbance at 450 nm and 550 nm. Readings at 550 nm were subtracted from those at 450 nm for correction purposes. A standard curve from recombinant mouse protein was constructed according to the manufacturer’s instructions to determine protein levels from the corrected optical densities using Deltasoft.

### Western blot analysis of local CD163 expression

Primary tumor and mammary gland lysates were equally loaded at a concentration of 20 μg protein per lane on a 12% polyacrylamide gel (GE Healthcare, Buckinghamshire, UK). Proteins were separated and transferred to a 0.45 μm nitrocellulose membrane (Bio-Rad). The membrane was incubated for 1 h at RT with blocking buffer (TBS supplemented with 0.1% Tween-20 and 5% non-fat dry milk) and subsequently with a primary rabbit anti-mouse CD163 monoclonal antibody (clone EPR19518, Abcam, diluted 1:1000 in blocking buffer) overnight at 4 °C. The membrane was then washed 3 times (5 min for each step) at RT with blocking buffer and incubated with a secondary donkey anti-rabbit IgG horseradish peroxidase (HRP) conjugated polyclonal antibody (Thermo Fisher Scientific, diluted 1:100,000 in blocking buffer) for 1 h at RT. The membrane was again washed 3 times (5 min for each step) at RT with blocking buffer and 1 time with destilled H_2_O (5 min) at RT before detection of the protein bands. For loading control, the membrane was incubated overnight at 4 °C with a primary rabbit anti-mouse glyceraldehyde 3-phosphate dehydrogenase (GAPDH) monoclonal antibody (clone EPR16891, Abcam, diluted 1:5000 in blocking buffer). Donkey anti-rabbit IgG HRP-conjugated polyclonal antibody (Thermo Fisher Scientific, diluted 1:2000 in blocking buffer) was used as secondary antibody to detect the protein bands. Visualization of the protein bands was performed on a ChemiDoc MP Imaging System (Bio-Rad) using a SuperSignal West Femto Kit (Thermo Fisher Scientific). All incubation steps were performed on a shaking platform. Precision Plus Protein Dual Color Standards (Bio-Rad) were loaded on the blot to identify the molecular weight of the CD163 and GAPDH protein bands. Quantitative analyses were performed with ImageJ.

### Statistical analyses

Statistical analyses were performed using SPSS Statistics 23 (IBM Analytics) and Prism (GraphPad). Data were checked for normality and, if necessary, log_10_ transformation was performed to normalize the data. Analysis of Variance (ANOVA) tests (with Tukey and Tamhane’s T2 post-hoc tests) and unpaired Student’s t-tests were applied to calculate *p*-values and determine whether differences between inoculation groups were statistically significant (*P* < 0.05).

## Results

### RAW264.7 macrophages increase ductal breakthrough, metastasis and splenomegaly but do not affect primary tumor growth of intraductally inoculated 4T1 cells

**Fig. 1:**
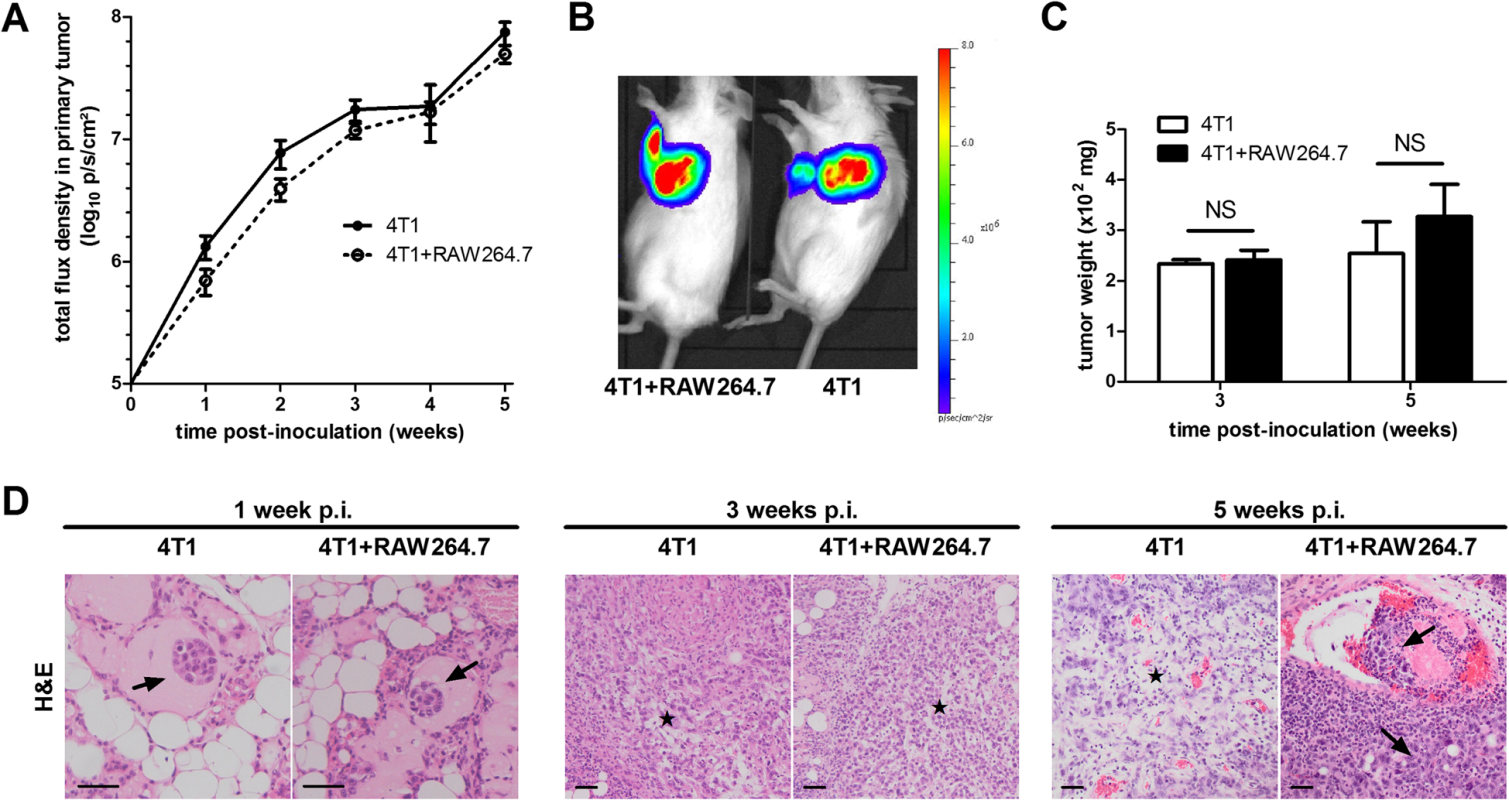
Similar primary tumor growth in 4T1 + RAW264.7 versus 4T1 intraductally inoculated mice. Lactating immunocompetent BALB/c mice were intraductally inoculated in the third mammary gland pair with bioluminescent traceable 4T1 mammary tumor cells either with or without RAW264.7 macrophages. **a** Primary tumor growth up to 5 w p.i. based on weekly measurements of the total flux radiance (p/s/cm^2^) at the inoculation sites (number of tumors at each time point: 4T1 + RAW264.7 inoculation group: *n* = 36 at 1 w p.i., *n* = 32 at 2 w p.i., *n* = 31 at 3 w p.i., *n* = 18 at 4 w p.i., *n* = 17 at 5 w p.i.; 4T1 inoculation group: *n* = 32 at 1 w p.i., *n* = 28 at 2 w p.i., *n* = 26 at 3 w p.i., *n* = 11 at 4 w p.i., *n* = 16 at 5 w p.i.). **b** Representative image of the bioluminescence signal at 5 w p.i. in both inoculation groups. **c** Measurement of the primary tumor weight at 3 and 5 w p.i. in both inoculation groups (number of tumors: 4T1 + RAW264.7 inoculation group: *n* = 8 at 3 w p.i., *n* = 14 at 5 w p.i.; 4T1 inoculation group: *n* = 8 at 3 w p.i., *n* = 10 at 5 w p.i.) **d** H&E histology of 4T1 + RAW264.7 and 4T1 primary tumor tissue at 1, 3 and 5 w p.i. Arrows indicate tumor cells; asterisks (*) indicate zones in the tumor mass that are characteristic for epithelial-to-mesenchymal transition (EMT). All scale bars = 50 μm. Data in panel (**a**) and (**c**) are presented as the means +/− standard error of the mean (SEM). NS: not significant

**Fig. 2:**
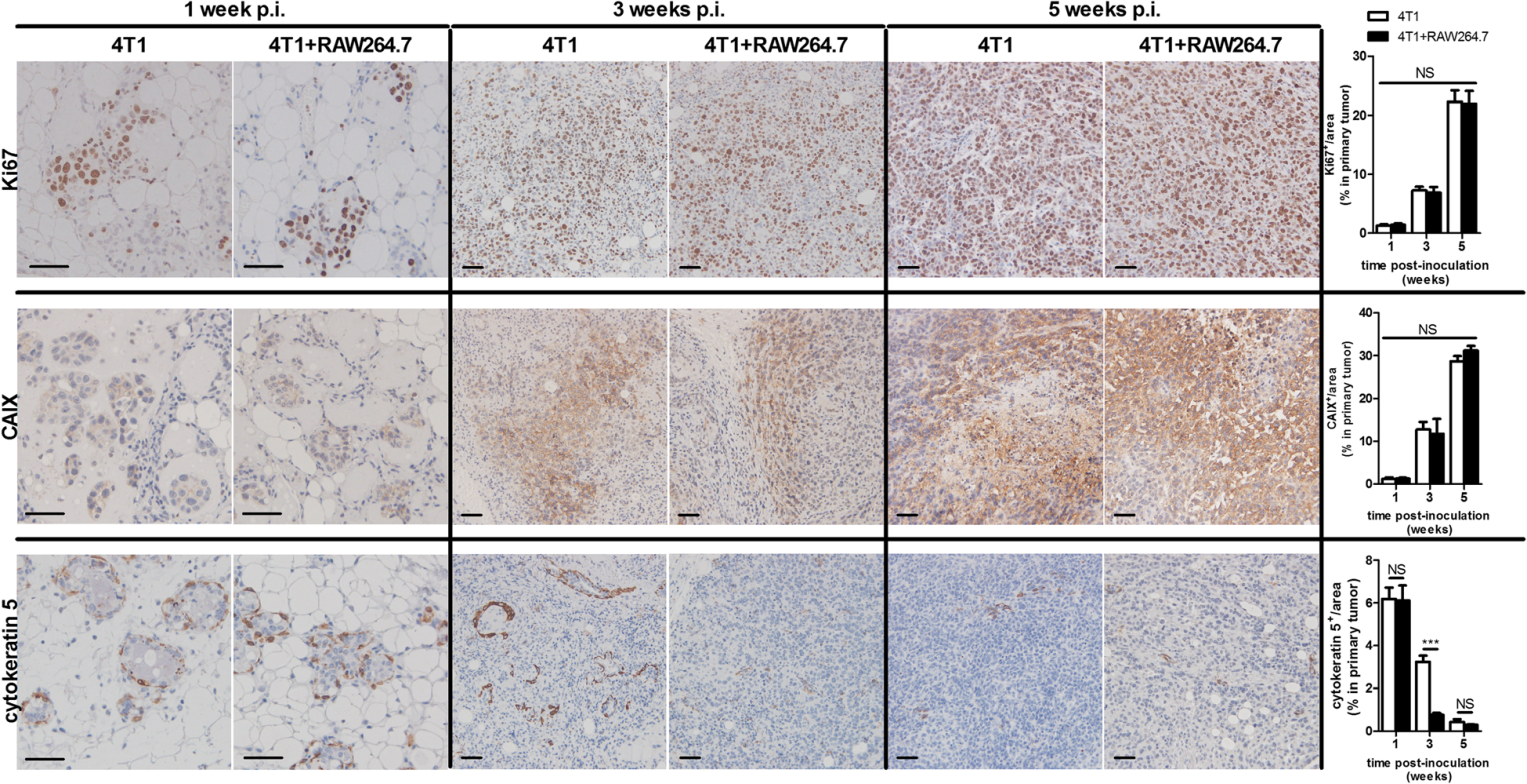
Similar cell proliferation and hypoxia, but differential ductal breakthrough in primary tumors of 4T1 + RAW264.7 versus 4T1 intraductally inoculated mice. Immunohistochemistry for the cell proliferation marker Ki67, hypoxia-associated enzyme carbonic anhydrase IX (CAIX) and myoepithelial cell marker cytokeratin 5 was performed on paraffin sections of primary tumors at 1, 3 and 5 w p.i. from 4T1 + RAW264.7 and 4T1 inoculated mice. The percentage of Ki67-, CAIX- and cytokeratin 5-positive staining was quantified (*n* = 5 at each time point and for each inoculation group). Scale bars = 50 μm. All data are presented as the means +/− SEM. NS: not significant, ***: *P* < 0.001

**Fig. 3:**
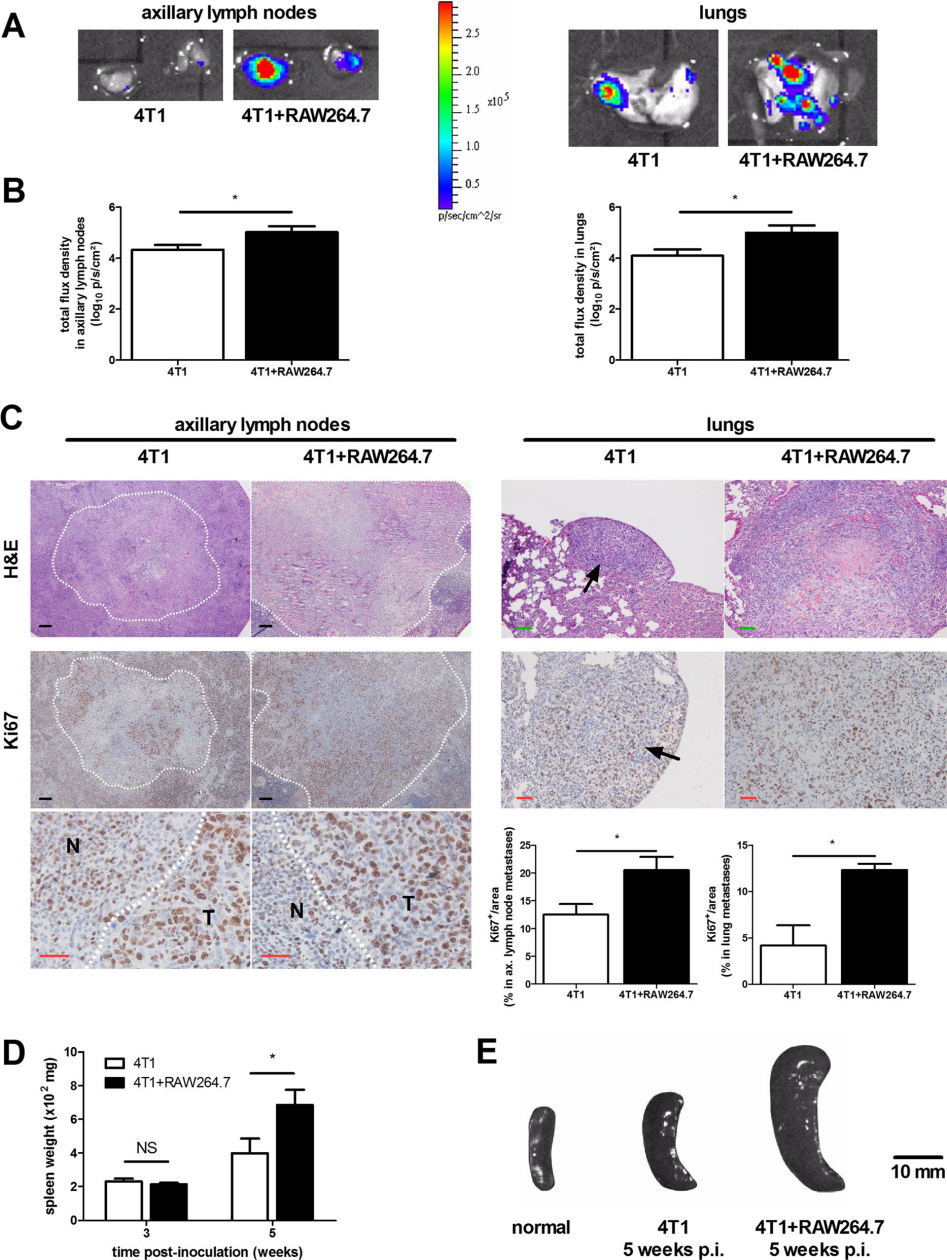
Differential metastasis and splenomegaly in 4T1 + RAW264.7 versus 4T1 intraductally inoculated mice. **a** Representative image of the ex vivo bioluminescence imaging of 4T1 metastases in isolated axillary lymph nodes and lungs at 5 w p.i. **b** Quantification of 4T1-derived bioluminescence signals (in p/s/cm^2^) in the axillary lymph nodes and lungs of 4T1 + RAW264.7 and 4T1 inoculated mice (4T1 + RAW264.7 inoculation group: *n* = 7 axillary lymph nodes, *n* = 8 lungs; 4T1 inoculation group: *n* = 8 axillary lymph nodes, *n* = 7 lungs). **c** H&E histology and Ki67 immunohistochemistry of axillary lymph node and lung metastases in 4T1 + RAW264.7 and 4T1 inoculated mice at 5 w p.i. White dashed lines and black arrows indicate tumor-rich regions. The detailed image of Ki67 staining in the axillary lymph nodes identifies the border between normal lymph node tissue (N) and tumor-rich tissue (T). The bar graphs show the percentage of Ki67-positive staining in the axillary lymph node and lung metastases from 4T1 + RAW264.7 and 4T1 inoculated mice (*n* = 5 axillary lymph nodes and *n* = 3 lungs for each inoculation group). Black scale bars = 200 μm, green scale bars = 100 μm, red scale bars = 50 μm. **d** Weight measurement of spleens isolated at 3 and 5 w p.i. from 4T1 + RAW264.7 and 4T1 inoculated mice (number of spleens: 4T1 + RAW264.7 inoculation group: *n* = 5 at 3 w p.i., *n* = 7 at 5 w p.i.; 4T1 inoculation group: *n* = 5 at 3 w p.i., *n* = 6 at 5 w p.i.). **e** Representative images of isolated spleens at 5 w p.i. from 4T1 + RAW264.7 and 4T1 inoculated mice. The image of an isolated spleen from a non-inoculated mouse is also shown for comparative purposes. Data in panel (**b**), (**c**) and (**d**) are presented as the means +/− SEM. NS: not significant, *: *P* < 0.05

To investigate the influence of macrophages and the macrophage-tumor cell crosstalk on primary tumor growth and ductal breakthrough 4T1 mammary tumor cells were intraductally inoculated with or without RAW264.7 macrophages (4T1 + RAW264.7 and 4T1 inoculation group, respectively) in lactating immunocompetent mice. Compared to 4T1 inoculated mice, the 4T1 + RAW264.7 inoculated mice showed no significant difference in primary tumor growth up to 5 w post-inoculation (p.i.) (Fig. 1a and b). Primary tumor weight at 3 w p.i. (i.e. when primary tumors were palpable, but no distant metastases were detectable) and 5 w p.i. (i.e. when metastases became apparent) in both the 4T1 and 4T1 + RAW264.7 inoculation group corroborated the similar primary tumor growth kinetics in both inoculation groups (Fig. 1c). Histology of the primary tumors at 1, 3 and 5 w p.i. indicated the invasiveness of the 4T1 cells in the lactating mammary ducts with tumor cells displaying characteristics of metastasis-preceding epithelial-to-mesenchymal transition (EMT) at 3 w p.i. and tumor cell invasion of blood vessels at 5 w p.i. (Fig. 1d). Ki67 staining qualitatively and quantitatively confirmed a similar tumor cell proliferation in both inoculation groups at 1, 3 and 5 w p.i. (Fig. 2). Stainings for carbonic anhydrase IX (CAIX), an enzyme that is upregulated in hypoxic tumors [17–19], allowed to investigate tumor hypoxia and identified a similar progressive increase in CAIX expression from 1 to 5 w p.i. in primary tumors from both inoculation groups (Fig. 2). Disruption of the ductal architecture by the tumor cells was quantitated based on cytokeratin 5 stainings for myoepithelial cells in primary tumors of 4T1 + RAW264.7 and 4T1 inoculated mice. Cytokeratin 5 positivity progressively decreased in the primary tumors and was significantly lower at 3 w p.i. in the 4T1 + RAW264.7 compared to the 4T1 tumors, indicative for enhanced tumor cell breakthrough in the presence of additional macrophages (Fig. 2). In order to evaluate distant metastases of 4T1 primary tumors both with and without RAW264.7 macrophage co-inoculation, the axillary lymph nodes and the lungs - typically the first sites to be affected by metastatic 4T1 cells - were isolated at 5 w p.i. and screened through ex vivo bioluminescence imaging. Both sites had traces of metastases (Fig. 3a). The liver was also isolated and screened but showed no traces of metastases (data not shown). Quantification of the bioluminescent signals identified increased metastasis in axillary lymph nodes and lungs of the 4T1 + RAW264.7 inoculation group (mean signal of 5.022 log_10_ ± 0.2359 p/s/cm^2^ in axillary lymph nodes and 5.003 log_10_ ± 0.2957 p/s/cm^2^ in lungs) compared to the 4T1 inoculation group (mean signal of 4.331 log_10_ ± 0.1972 p/s/cm^2^ in axillary lymph nodes and 4.097 log_10_ ± 0.2519 p/s/cm^2^ in lungs) (Fig. 3b). Comparative H&E and Ki67 stainings of axillary lymph node and lung sections from metastasis-bearing mice in the 4T1 + RAW264.7 versus 4T1 inoculation group corroborated the ex vivo bioluminescence imaging results with enhanced metastatic tumor burden and tumor cell proliferation in the 4T1 + RAW264.7 inoculation group (Fig. 3c). The lung metastatic growth in both inoculation groups was further characterized immunohistochemically by staining of the immune cell infiltrates (identified by immune cell markers CD45 (pan-immune cell marker), Ly6G (neutrophil marker), CD8a (cytotoxic immune cell marker) and CD163 (anti-inflammatory macrophage marker)) and vascular structures (identified by the endothelial cell marker CD31) (Additional file 1: Figure S1).

The advancement in primary tumor growth and metastasis was reflected by a progressive splenomegaly in both inoculation groups. Whereas the spleen weight did not differ at 3 w p.i. between the 4T1 + RAW264.7 and 4T1 inoculation group, splenomegaly became more pronounced at 5 w p.i. in the 4T1 + RAW264.7 inoculation group (Fig. 3d and e). Indeed, spleen weights and sizes at 5 w p.i. were approximately 1.5 times larger in the 4T1 + RAW264.7 inoculation group compared to the 4T1 inoculation group.

### Co-inoculation of RAW264.7 macrophages with 4T1 mammary tumor cells mediates a M1 to M2 macrophage polarization in the mammary tumor microenvironment

**Fig. 4:**
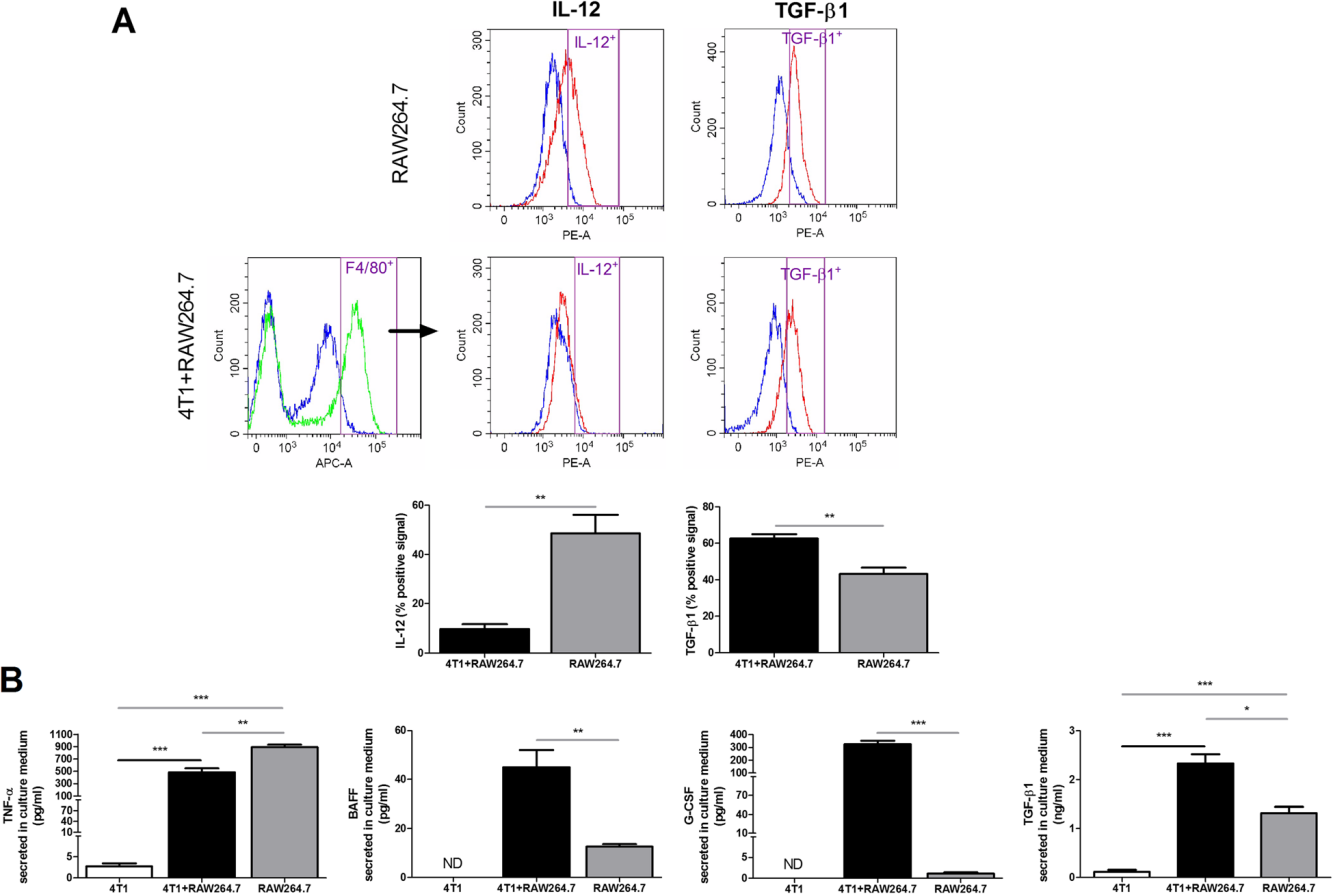
M1 to M2 polarization upon co-culturing of RAW264.7 macrophages with 4T1 tumor cells. **a** Flow cytometric analysis for intracellular expression of the pro-inflammatory/M1-related cytokine IL-12 and anti-inflammatory/M2-related cytokine TGF-β1 in RAW264.7 macrophages after 96 h mono-culture and co-culture with 4T1 tumor cells (*n* = 3 for all culture conditions). RAW264.7 macrophages derived from 4T1 + RAW264.7 co-cultures were gated (purple) based on positivity for F4/80 (green line) relative to the isotype control (blue line). The gates for IL-12 and TGF-β1 (purple) were applied for quantification of both positive signals (red line) relative to their isotype control signal (blue line). The bar graphs show the percentage of the F4/80-positive cells based on the applied gatings. **b** Levels of secreted pro-inflammatory/M1-related (TNF-α) and anti-inflammatory/M2-related (BAFF, G-CSF and TGF-β1) cytokines in cell-free culture media of 24 h mono- and co-cultures of RAW264.7 macrophages and 4T1 mammary tumor cells (*n* = 3 for all culture conditions). All data are presented as the means +/− SEM. ND: not detectable, *: *P* < 0.05, **: *P* < 0.01, ***: *P* < 0.001

**Fig. 5:**
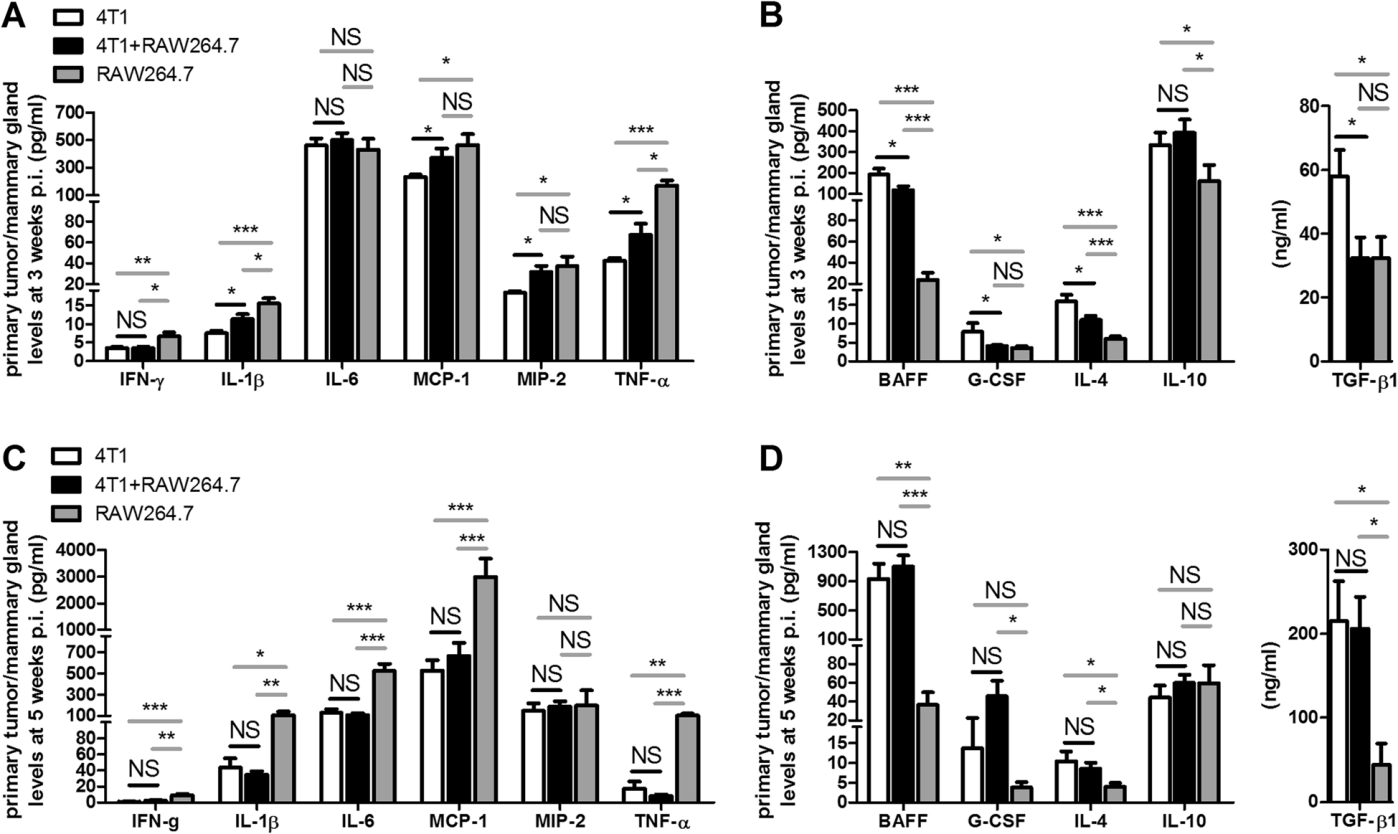
M1 to M2 polarization upon disease progression in 4T1 + RAW264.7, 4T1 and RAW264.7 intraductally inoculated mice. **a**, **b** Pro-inflammatory/M1-related (IFN-γ, IL-1β, IL-6, MCP-1, MIP-2 and TNF-α) (**a**) and anti-inflammatory/M2-related cytokine levels (BAFF, G-CSF, IL-4, IL-10 and TGF-β1) (**b**) at 3 w p.i. in primary tumors/mammary glands from 4T1 + RAW264.7, 4T1 and RAW264.7 inoculated mice (*n* = 6 tumors/mammary glands for each inoculation group). **c**, **d** Pro-inflammatory/M1-related (**c**) and anti-inflammatory/M2-related cytokine levels (**d**) at 5 w p.i. in primary tumors/mammary glands from 4T1 + RAW264.7, 4T1 and RAW264.7 inoculated mice (4T1 + RAW264.7 inoculation group: *n* = 10 tumors; 4T1 inoculation group: *n* = 7 tumors; RAW264.7 inoculation group: *n* = 6 mammary glands). All data are presented as the means +/− SEM. NS: not significant, *: *P* < 0.05, **: *P* < 0.01, ***: *P* < 0.001

Macrophages are known as important immunomodulators in the breast tumor microenvironment and can strongly affect disease progression. More specifically, macrophages can stimulate either pro- or anti-inflammatory immune responses based on their activation state, i.e. either M1 or M2. Flow cytometric analysis identified a M1 to M2 polarization of RAW264.7 macrophages co-cultured with 4T1 tumor cells based on their significantly decreased intracellular expression of the M1-related (i.e. pro-inflammatory) cytokine interleukin (IL)-12 and significantly increased intracellular expression of the M2-related (i.e. anti-inflammatory) cytokine transforming growth factor (TGF)-β1 compared to RAW264.7 macrophages that were cultured alone (Fig. 4a). In addition, culture media of 4T1 + RAW264.7 co-cultures showed significantly decreased levels of the M1-related cytokine tumor necrosis factor (TNF)-α and significantly increased levels of the M2-related cytokines B-cell activating factor (BAFF), granulocyte colony-stimulating factor (G-CSF) and TGF-β1 compared to RAW264.7 mono-culture media (Fig. 4b). To provide more insights into the immunological impact of the co-inoculated macrophages on the observed tumor progression in vivo, local and systemic M1−/M2-related cytokine profiles were investigated and compared at 3 and 5 w p.i. in the primary tumor lysates and sera from the 4T1 + RAW264.7 and 4T1 inoculation group. At 3 w p.i. 4T1 + RAW264.7 primary tumor lysates showed a significant increase in the M1-related cytokines IL-1β, monocyte chemoattractant protein (MCP)-1, macrophage inflammatory protein (MIP)-2 and TNF-α compared to the 4T1 primary tumors, whereas the M2-related cytokines BAFF, G-CSF, IL-4 and TGF-β1 showed significantly lower levels in the 4T1 + RAW264.7 versus the 4T1 primary tumors (Fig. 5a and b). However, at 5 w p.i. these differences in M1- and M2-related cytokine profiles could no longer be detected between both inoculation groups (Fig. 5c and d). M1- and M2-related cytokines were also measured in lysates of RAW264.7 macrophage-only intraductally inoculated mammary glands. These results showed that the 4T1 tumor cells strongly influence the local and RAW264.7 macrophage-mediated immune responses as, compared to 4T1 + RAW264.7 and 4T1 primary tumors, RAW264.7-only mammary glands contained higher M1-related cytokine levels (interferon (IFN)-γ, IL-1β and TNF-α at 3 and 5 w p.i., and MCP-1 and IL-6 at 5 w p.i. compared to 4T1 + RAW264.7 and 4T1; MCP-1 and MIP-2 at 3 w p.i. compared to 4T1) and lower M2-related cytokine levels (BAFF and IL-4 at 3 and 5 w p.i., IL-10 at 3 w p.i., and TGF-β1 at 5 w p.i. compared to 4T1 + RAW264.7 and 4T1; G-CSF and TGF-β1 at 3 w p.i. compared to 4T1; G-CSF at 5 w p.i. compared to 4T1 + RAW264.7) (Fig. 5).

In contrast to the local primary tumor the serum cytokine levels were far less different between the 4T1 + RAW264.7 and 4T1 inoculation group at 3 and 5 w p.i. More specifically, at 3 w p.i. the serum levels of the M1-related cytokine MCP-1 were higher and the M2-related TGF-β1 serum levels were lower in the 4T1 + RAW264.7 compared to the 4T1-only inoculation group (Additional file 2: Figure S2A and B). These systemic differences were no longer detected at 5 w p.i. (Additional file 2: Figure S2C and D). In contrast to tumor-bearing mice, serum cytokine profiles at 3 and 5 w p.i. in the RAW264.7 inoculation group showed increased levels of M1-related cytokines (MCP-1 and MIP-2 at 3 and 5 w p.i., and TNF-α at 5 w p.i. compared to 4T1 + RAW264.7 and 4T1) and decreased levels of M2-related cytokines (BAFF at 3 and 5 w p.i., and G-CSF at 5 w p.i. compared to 4T1 + RAW264.7 and 4T1; TGF-β1 at 3 and 5 w p.i. compared to 4T1), reflecting the local M1−/M2-related cytokine profile differences (Additional file 2: Figure S2).

**Fig. 6:**
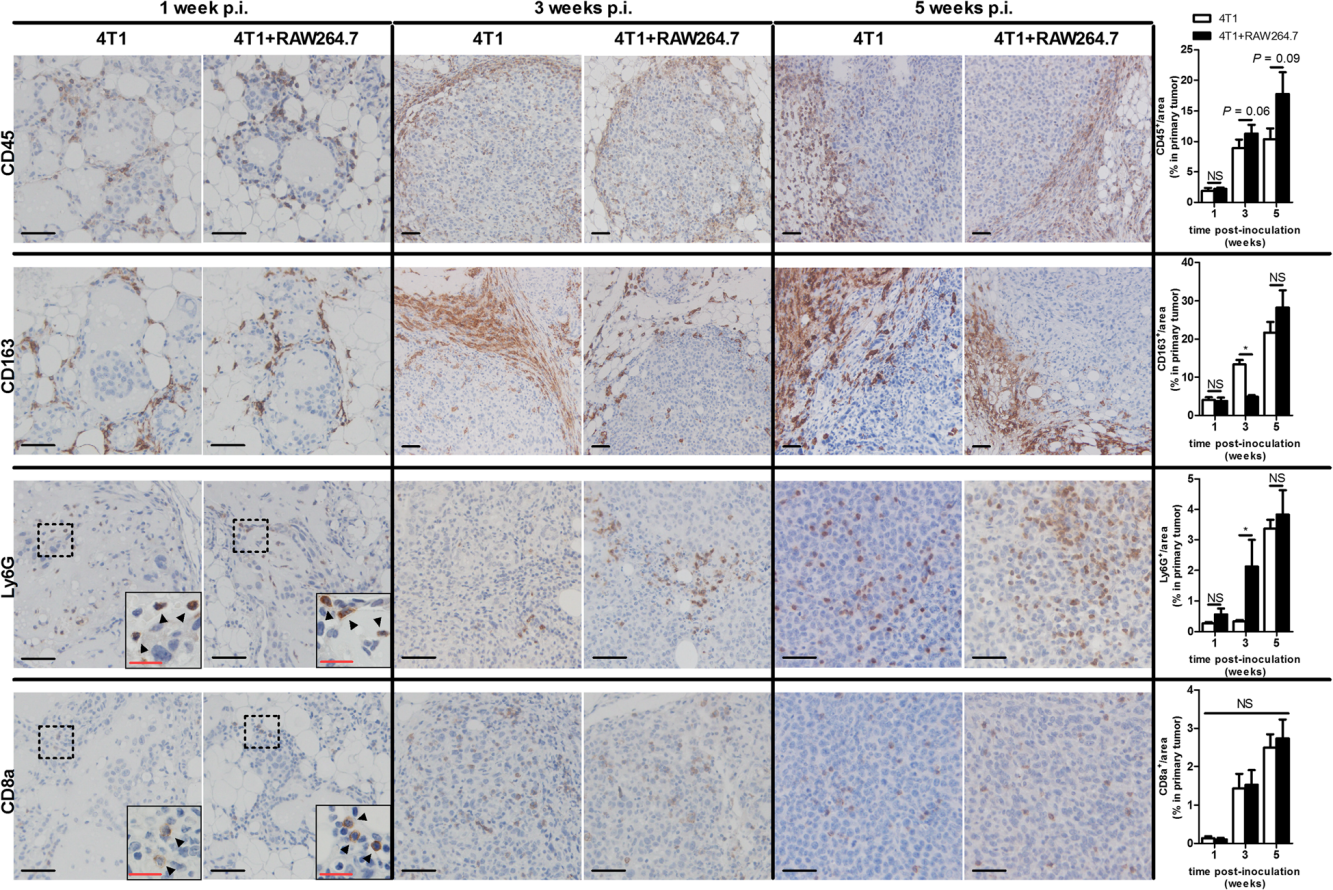
Immunohistochemistry of the tumor immune microenvironment in 4T1 + RAW264.7 versus 4T1 intraductally inoculated mice. Immunohistochemistry for immune cell markers CD45 (pan-immune cell marker), CD163 (anti-inflammatory macrophage marker), Ly6G (neutrophil marker) and CD8a (cytotoxic T-cell marker) was performed on paraffin sections of primary tumors at 1, 3 and 5 w p.i. from 4T1 + RAW264.7 and 4T1 inoculated mice. The percentage of CD45-, CD163-, Ly6G- and CD8a-positive staining was quantified (*n* = 5 at each time point and for each inoculation group). Dashed inserts and arrowheads highlight the few Ly6G- and CD8a-positive cells at 1 w p.i. Black scale bars = 50 μm; red scale bars = 20 μm. All data are presented as the means +/− SEM. NS: not significant, *: *P* < 0.05

In order to further investigate the immunological profile of the primary tumors in both the 4T1 + RAW264.7 and 4T1 inoculation group, immunohistochemistry for CD45, CD163, Ly6G and CD8a was performed on primary tumor sections at pre- (1 and 3 w p.i.) and metastatic (5 w p.i.) time points (Fig. 6). Based on the pan-immune cell marker CD45, the percentage of immune cells increased and these cells invaded both the 4T1 + RAW264.7 and 4T1 primary tumors over time, but also remained partly in the surrounding stroma (Fig. 6). At 1 w p.i., when tumor cells were still in situ and did not yet invade the mammary fat pad, immune cells were scarce. Only few CD163-positive cells could be detected, which mainly comprise tumor-recruited anti-inflammatory/M2 macrophages. The CD163 stainings could not detect the intraductally inoculated RAW264.7 macrophages as these cells are initially M1 polarized and also do not express CD163 based on immunohistochemistry and western blotting for CD163, respectively on paraffin slides and in lysates of RAW264.7 inoculated mammary glands (Additional file 3: Figure S3). At 3 w p.i. the CD163-positive tumor-recruited cells remained outside the tumor area, but strongly increased in the 4T1 inoculation group whereas in the 4T1 + RAW264.7 inoculation group a similar CD163-positive staining was observed at 3 w p.i. compared to 1 w p.i (Fig. 6). At 5 w p.i. the 4T1 + RAW264.7 and 4T1 primary tumors showed similar CD163 stainings, but the CD163-positive cells still minimally invaded the tumor tissue. Western blotting for CD163 corroborated the lower CD163 expression at 3 w p.i. and similar CD163 expression at 5 w p.i in 4T1 + RAW264.7 primary tumors compared to 4T1 primary tumors (Additional file 3: Figure S3B). Additional immunohistochemistry for Ly6G and CD8a in 4T1 + RAW264.7 and 4T1 primary tumors also showed few staining around the tumor-harboring mammary ducts at 1 w p.i. However, at 3 and 5 w p.i. the Ly6G and CD8a positivity increased and, in marked contrast to CD163, were located within the primary tumor area. Compared to the 4T1 primary tumors, 4T1 + RAW264.7 primary tumors qualitatively and quantitatively displayed increased staining for Ly6G at 3 w p.i., whereas similar staining was detected in both inoculation groups for CD8a at that time point, and for Ly6G and CD8a stainings at 5 w p.i (Fig. 6).

**Fig. 7:**
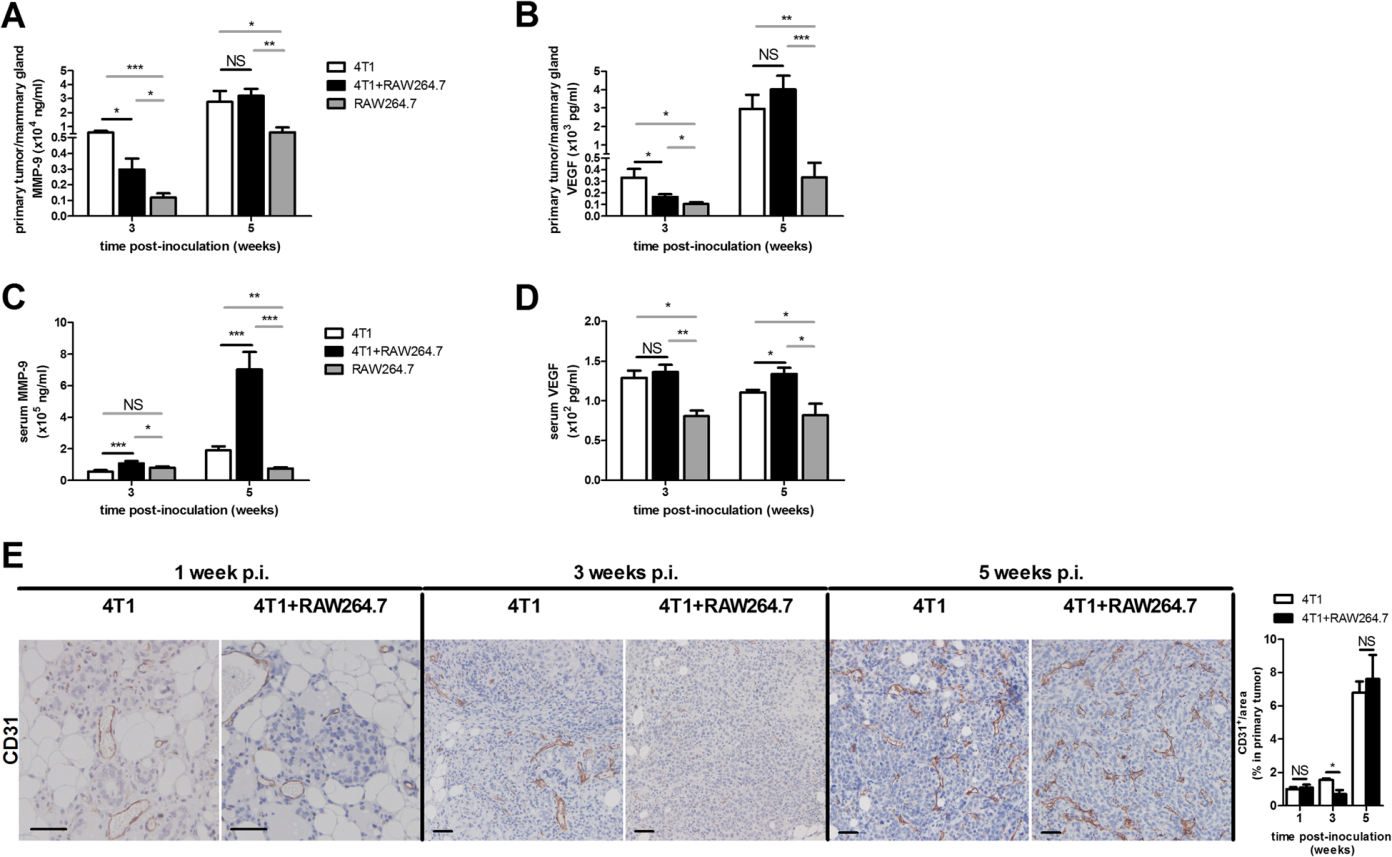
Local and systemic MMP-9 and VEGF in 4T1 + RAW264.7, 4T1 and RAW264.7 intraductally inoculated mice. **a**, **b** Primary tumor/mammary gland MMP-9 (**a**) and VEGF levels (**b**) at 3 and 5 w p.i. in 4T1 + RAW264.7, 4T1 and RAW264.7 inoculated mice (4T1 + RAW264.7 inoculation group: *n* = 8 tumors at 3 w p.i. and *n* = 12 tumors at 5 w p.i.; 4T1 inoculation group: *n* = 8 tumors at 3 w p.i. and *n* = 6 tumors at 5 w p.i.; RAW264.7 inoculation group: *n* = 8 mammary glands at 3 w p.i. and *n* = 7 mammary glands at 5 w p.i.). **c**, **d** Serum MMP-9 (**c**) and VEGF levels (**d**) at 3 and 5 w p.i. in 4T1 + RAW264, 4T1 and RAW264.7 inoculated mice (4T1 + RAW264.7 and 4T1 inoculation group: *n* = 6 sera at 3 and 5 w p.i.; RAW264.7 inoculation group: *n* = 4 sera at 3 and 5 w p.i.). **e** CD31 immunohistochemistry on primary tumor sections from 4T1 + RAW264.7 and 4T1 inoculated mice at 1, 3 and 5 w p.i. (*n* = 5 at each time point and for each inoculation group). All scale bars = 50 μm. All data are presented as the means +/− SEM. NS: not significant, *: *P* < 0.05, **: *P* < 0.01, ***: *P* < 0.001

Extracellular matrix degradation and angiogenesis were investigated at the protein level by measuring the primary tumor lysate and serum levels of MMP-9 and VEGF in the 4T1 + RAW264.7 and the 4T1 inoculation group at 3 and 5 w p.i. Local MMP-9 and VEGF levels in the primary tumor lysates increased as tumor growth progressed from 3 to 5 w p.i. (Fig. 7a and b). However, at 3 w p.i. the MMP-9 and VEGF levels significantly decreased in the 4T1 + RAW264.7 compared to the 4T1 inoculation group, whereas this difference was no longer seen at 5 w p.i. (Fig. 7a and b). In accordance with the local and systemic cytokine profiles, significantly decreased MMP-9 and VEGF levels were detected in the RAW264.7 inoculated mammary gland lysates compared to the 4T1 + RAW264.7 and 4T1 inoculated counterparts at 3 and 5 w p.i. (Fig. 7a and b). Systemic MMP-9 levels also progressively increased over time and were significantly higher at both 3 and 5 w p.i. in the 4T1 + RAW264.7 inoculation group compared to the 4T1 inoculation group and significantly lower in the RAW264.7 inoculation group at 3 w p.i. compared to the 4T1 + RAW264.7 inoculation group and at 5 w p.i. compared to both the 4T1 + RAW264.7 and 4T1 inoculation group (Fig. 7c). In contrast, VEGF serum levels were close to the detection limit and moreover did not increase between 3 and 5 w p.i. (Fig. 7d). Whereas the systemic VEGF levels in the 4T1 + RAW264.7 and 4T1 inoculation group did not differ at 3 w p.i., at 5 w p.i. the 4T1 + RAW264.7 inoculation group showed significantly higher systemic VEGF levels compared to the 4T1 inoculation group (Fig. 7d). Systemic VEGF levels in the RAW264.7 inoculation group were significantly lower compared to the 4T1 + RAW264.7 and 4T1 inoculation group at both 3 and 5 w p.i. (Fig. 7d).

Immunohistochemical stainings of 4T1 + RAW264.7 and 4T1 primary tumors with the endothelial cell marker CD31 were indicative for an increase in vascular growth over time. Upon quantification, significantly less CD31 staining was observed in 4T1 + RAW264.7 compared to 4T1 tumors upon invasion of tumor cells in the mammary fat pad at 3 w p.i., mirroring the difference in primary tumor VEGF response at that early time point (Fig. 7e).

### Onco-immunological responses and metastasis after intraductal co-inoculation of 4T1 mammary tumor cells with RAW264.7 macrophages correlate with local and systemic CHI3L1 and LCN2 levels

**Fig. 8:**
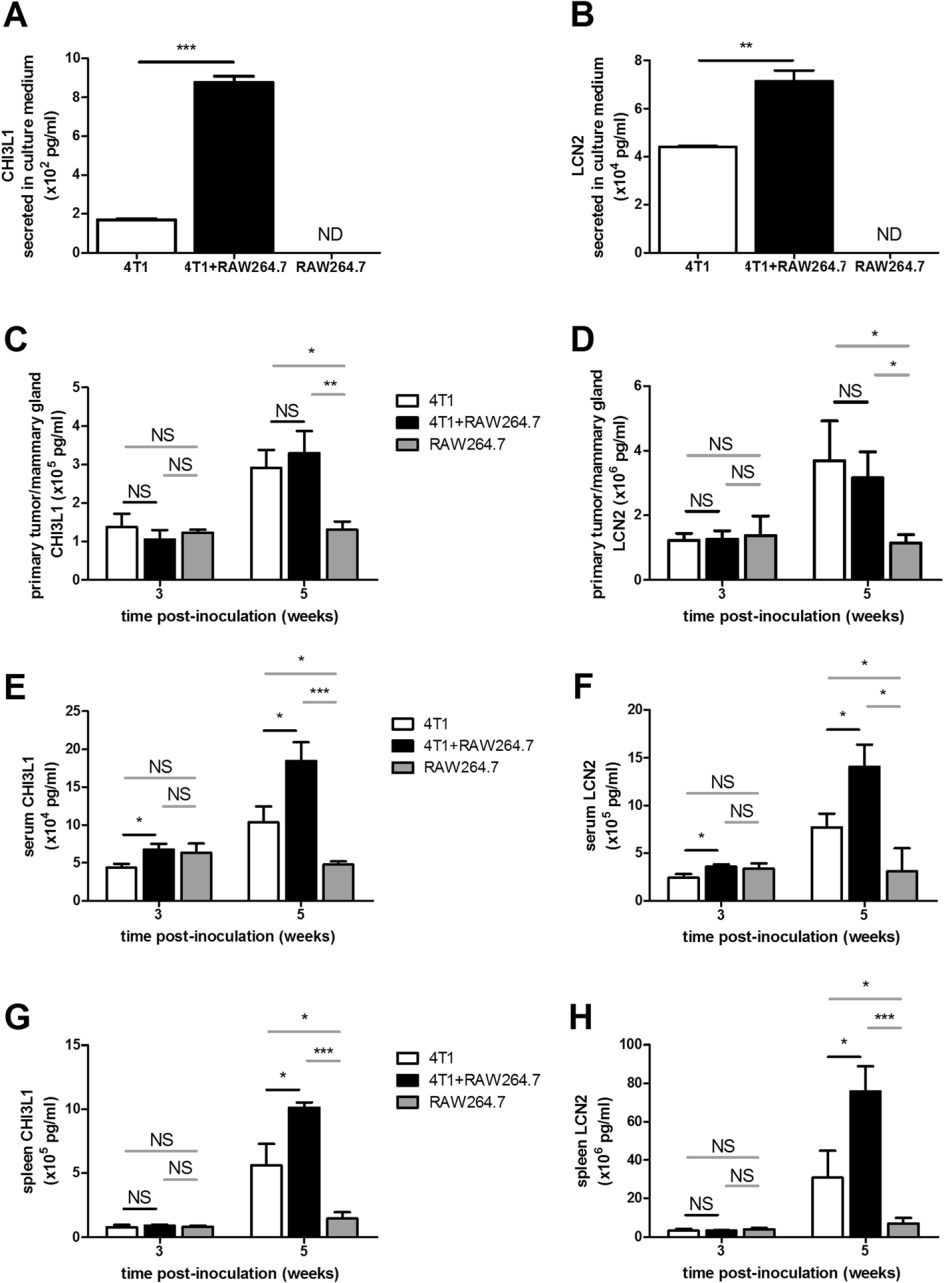
CHI3L1 and LCN2 as immune-associated disease biomarkers in 4T1 + RAW264.7, 4T1 and RAW264.7 intraductally inoculated mice. **a**, **b** CHI3L1 (**a**) and LCN2 (**b**) levels secreted in cell-free culture media of 24 h mono- and co-cultures of 4T1 mammary tumor cells and RAW264.7 macrophages (*n* = 3 for all culture conditions). **c**, **d** Primary tumor/mammary gland CHI3L1 (**c**) and LCN2 levels (**d**) at 3 and 5 w p.i. in 4T1 + RAW264.7, 4T1 and RAW264.7 inoculated mice (4T1 + RAW264.7 inoculation group: *n* = 8 tumors at 3 w p.i. and *n* = 12 tumors at 5 w p.i.; 4T1 inoculation group: *n* = 8 tumors at 3 w p.i. and *n* = 6 tumors at 5 w p.i.; RAW264.7 inoculation group: *n* = 5 mammary glands at 3 and 5 w p.i.). **e**, **f** Serum CHI3L1 (**e**) and LCN2 levels (**f**) at 3 and 5 w p.i. in 4T1 + RAW264.7, 4T1 and RAW264.7 inoculated mice (4T1 + RAW264.7 inoculation group: *n* = 6 sera at 3 w p.i. and *n* = 7 sera at 5 w p.i.; 4T1 inoculation group: *n* = 6 sera at 3 and 5 w p.i.; RAW264.7 inoculation group: *n* = 4 sera at 3 and 5 w p.i.). **g**, **h** Splenic CHI3L1 (**g**) and LCN2 levels (**h**) at 3 and 5 w p.i. in 4T1 + RAW264.7, 4T1 and RAW264.7 inoculated mice (*n* = 5 at each time point and for each inoculation group). All data are presented as the means +/− SEM. NS: not significant, *: *P* < 0.05, **: *P* < 0.01, ***: *P* < 0.001

CHI3L1 and LCN2 have previously been identified as immune-related biomarkers for disease outcome in breast cancer patients as well as for monitoring tumor progression in the intraductal mouse model for TNBC [6, 12–14]. Measurement of these two parameters in 4T1 + RAW264.7 and 4T1-only tumor-bearing mice could therefore verify the observed differential breast cancer burden between the two inoculation groups. Preliminary in vitro tests showed that co-cultures of 4T1 tumor cells with RAW264.7 macrophages secreted higher amounts of both CHI3L1 and LCN2 than either 4T1 or RAW264.7 mono-cultures (Fig. 8a and b). CHI3L1 and LCN2 levels were subsequently measured in primary tumor lysates and sera from 4T1 + RAW264.7 and 4T1 inoculated mice and showed a progressive increase from 3 to 5 w p.i. (Fig. 8c-f). Local levels did not differ between 4T1 + RAW264.7 and 4T1 primary tumors, which is indicative for the similar primary tumor growth observed in both inoculation groups (Fig. 8c and d). In marked contrast, systemic levels significantly increased in 4T1 + RAW264.7 inoculated mice, mirroring the increased CHI3L1 as well as LCN2 secretion found in 4T1 + RAW264.7 co-cultures (Fig. 8e and f). Local and systemic levels in the RAW264.7 inoculation group were also lower than those in the 4T1 + RAW264.7 and 4T1 counterparts at 5 w p.i., yet not at 3 w p.i. (Fig. 8c-f). Additional measurements of the CHI3L1 and LCN2 levels in spleen lysates from 4T1 + RAW264.7 and 4T1 inoculated mice showed that in the two inoculation groups both biomarkers increased over time with enhanced splenomegaly, but remained low in the RAW264.7-only inoculated mice. At 5 w p.i., when spleen sizes in the 4T1 + RAW264.7 inoculation group were more severely increased compared to their 4T1 counterparts, CHI3L1 and LCN2 levels were also significantly higher in the 4T1 + RAW264.7 spleen lysates than in the 4T1 spleen lysates (Fig. 8g and h). Spleens from RAW264.7-only inoculated mice showed significantly decreased CHI3L1 and LCN2 levels at 5 w p.i. compared to both the 4T1 + RAW264.7 and 4T1 inoculation group (Fig. 8g and h).

## Discussion

Preclinical models remain essential for investigating novel therapies to tackle cancer in humans. In the case of breast cancer, such models are often established by grafting mammary tumor cells in the mouse mammary gland [20, 21]. Although the fat pad is the most frequently inoculated site for these cells, we and others have shown that the mammary ducts are a valuable alternative inoculation site [6, 22–28]. When comparing both inoculation sites, the fat pad model is representative for advanced invasive breast tumors whereas the intraductal model more closely resembles human breast cancer progression starting from DCIS before turning into invasive and metastatic breast cancer. In a previous study from our group, we observed that intraductally inoculated 4T1 tumor cells - which lack ER, PR and HER2 expression - show a slower primary tumor growth with similar metastasis compared to their fat pad inoculated counterparts [6]. Because of the lactating state of these mice, tumor growth in the intraductal model is accompanied by an involution process, resembling pregnancy-associated breast cancer [29, 30]. Therefore, the intraductal tumor model is also a relevant tool for investigating this rarely studied, aggressive breast cancer type. Furthermore, as immunocompetent mice were at first used, this allowed the study of tumor-immune cell interactions associated with ductal mammary tumor progression.

Since macrophages are among the most important regulators of the immune interactions in the breast tumor microenvironment [8, 31], the current study focused on immune responses upon their intraductal co-inoculation with 4T1 tumor cells. Either with or without additional macrophages, the tumor cells grew within the mammary ducts, invaded the ductal barrier and progressed in the mammary fat pad from which they spread towards the axillary lymph nodes and lungs in the presence of a leukocytic reaction. In both intraductal inoculation groups, primary tumors developed at a similar rate and displayed similar Ki67 tumor cell proliferation and hypoxia-associated CAIX expression. However, based on stainings for the myoepithelial cell marker cytokeratin 5 in primary tumors, the 4T1 cells disrupted the ductal structures faster in the presence of additional macrophages. Correlating with this enhanced ductal breakthrough and tumor cell invasion, metastases in axillary lymph nodes and lungs were increased in the mice co-inoculated with additional macrophages.

Interestingly, in both inoculation groups, lung metastases showed characteristics of vessel co-option, i.e. the metastasized tumor cells in the lung incorporated pre-existing alveolar blood vessels rather than inducing angiogenesis [32]. This non-angiogenic/alveolar histopathological growth pattern of 4T1 lung metastases has been reported with intravenous injections of 4T1 tumor cells and correlated with poor response to anti-angiogenic therapy [32]. In the current study, the tumor-bearing mice inoculated with additional macrophages also suffered from more severe splenomegaly, corroborating the reported increased splenic erythropoiesis which compensated for more advanced 4T1 tumor progression [33] and also pointed to an augmented leukemoid reaction [6, 34]. Overall, our results indicate that additional macrophages have a stimulatory effect on metastasis in the intraductal model for TNBC.

Upon association of macrophages with a tumor, they become TAM which stimulate breast cancer progression by eliciting an immune response in favor to the growing mammary tumor [7, 8]. This characteristic differentiates them from the original homeostatic tissue macrophages that in marked contrast have the ability to eliminate tumor cells through the establishment of a pro-inflammatory environment and the presentation of tumor antigens [7, 8]. These macrophages are classified as classically activated or M1-type macrophages. Indeed, tumor cells can influence these innate immune effectors by educating them and change their activation state towards M2 (i.e. alternatively activated macrophages) in order to establish an anti-inflammatory microenvironment that suppresses the host’s antitumor immune response and ultimately causes tumor immune evasion [7–9, 35, 36]. Our findings are highly indicative for a similar polarization of the RAW264.7 macrophage inoculum by tumor cell signaling in the intraductal model for TNBC. The decreased intracellular production of the M1-related cytokine IL-12 and increased intracellular production of the M2-related cytokine TGF-β1 by RAW264.7 macrophages co-cultured with 4T1 tumor cells compared to mono-cultured RAW264.7 macrophages confirmed the M1 to M2 polarization hypothesis, which was further corroborated by secreted cytokine measurements in the culture media. Turning to the in vivo situation, pre-metastatic 4T1 + RAW264.7 primary tumors showed enhanced pro-inflammatory/M1-related and decreased anti-inflammatory/M2-related cytokine profiles compared to 4T1 primary tumors, whereas these differences between both inoculation groups were no longer detectable upon metastasis. It has been shown that 4T1 tumor cells communicate with RAW264.7 macrophages resulting in the polarization of these immune cells and the subsequent production of an immune suppressing tumor microenvironment [37, 38]. In line with the education of the macrophages towards a M2 phenotype, the M1-related cytokine profile was lower and the M2-related cytokine profile was higher in primary tumors of 4T1 + RAW264.7 inoculated mice at 3 and 5 w p.i. compared to RAW264.7 inoculated mammary glands (i.e. no communication between 4T1 tumor cells and RAW264.7 macrophages). Based on intraductal inoculations with saline (sham) and the determination of the induced immune cell influx and cytokine profile as previously described by our group [39–42], it can be suggested that the intraductal inoculation method in se establishes a minimal local inflammation, which will have contributed to a limited extent to the substantial pro-inflammatory response observed in the primary tumors in the current study. It might be a concern that macrophages potentially create doublets with tumor cells and could migrate to seed what looks like metastatic spread [43, 44]. However, this scenario is unlikely to occur at early disease stages when the RAW264.7 inoculum shows characteristics of a M1 phenotype. Instead of doublet formation, such M1/tumoricidal macrophages will recognize the tumor cells as non-self and phagocytose them, resulting in the loss of tumor cell-derived luminescent signal [45]. On the other hand, tumor cell/macrophage doublet formation cannot be ruled out at later stages when M2 macrophage polarization occurs and RAW264.7 macrophages may lose their tumoricidal activity. Nevertheless, our results suggest that macrophage transfer and the proposed M1 to M2 macrophage polarization facilitated the metastasis of intraductal 4T1 tumor cells, complementing a previous study relying on mammary fat pad inoculations [46].

Immunohistochemical evaluation of pre- and post-metastatic primary tumors showed an increase in the recruitment of immune cells as a result of the cytokine responses. More specifically, the infiltration of CD8a-positive T-cells in both inoculation groups indicates that the 4T1-based intraductal model for TNBC gives rise to inflamed tumors [47], highlighting its relevance for future screening of novel candidate immunotherapeutics. Furthermore, the decreased staining of the anti-inflammatory/M2 macrophage marker CD163 and increased staining of the neutrophil marker Ly6G at 3 w p.i. in 4T1 + RAW264.7 tumors verified the observed respectively M2-related and M1-related cytokine profiles in 4T1 + RAW264.7 inoculated mice prior to metastasis.

Macrophages also support breast cancer metastasis by releasing matrix degrading MMPs such as MMP-9 and angiogenic proteins such as VEGF. In the current study, primary tumor MMP-9 and VEGF levels confirmed the macrophage polarization from M1 to M2 and establishment of an immune suppressing/tumor-supporting microenvironment in 4T1 + RAW264.7 inoculated mice as primary tumors from this inoculation group showed lower MMP-9 and VEGF levels compared to the 4T1 inoculation group at pre-metastasis, but similar levels upon metastasis. Our proposed M1 to M2 polarization was emphasized by the lower levels of both mediators in RAW264.7 inoculated mice and by CD31 immunohistochemistry. Moreover, the strong increase in serum MMP-9 and the moderate increase in VEGF serum levels in the 4T1 + RAW264.7 compared to 4T1 inoculated mice correlated with a significant increase in systemic metastasis of the 4T1 primary tumors.

For verification of the mammary tumor disease progression either with or without additional macrophages, two immune-related proteins CHI3L1 and LCN2, which both have been identified as prognostic biomarkers in breast cancer patients [12–14], were incorporated in the current study. Indeed, we previously showed that CHI3L1 and LCN2 levels increase progressively in primary tumors and sera of 4T1 intraductally inoculated mice and confirmed the increase in tumor-associated leukemoid responses as well as primary tumor growth and systemic metastasis detected by in vivo and ex vivo bioluminescence imaging [6]. Based on these observations, the in vitro observation of increased CHI3L1 and LCN2 secretion in 4T1 + RAW264.7 co-cultures compared to 4T1 and RAW264.7 mono-cultures provided a preliminary indication of enhanced onco-immunological responses due to the crosstalk between the tumor cells and macrophages. In our in vivo experiments, similar primary tumor levels but increased serum and spleen CHI3L1 and LCN2 levels were measured in 4T1 + RAW264.7 compared to 4T1 inoculated mice corresponding to the similar primary tumor growth in both inoculation groups, the increased metastasis and enhanced leukemoid reaction in the additional macrophages inoculation group. The lower local and systemic levels of both biomarker proteins in RAW264.7 inoculated mice at 5 w p.i. further indicated the critical importance of crosstalk with tumor cells and anti-inflammatory signaling for the induction of CHI3L1 as well as LCN2. CHI3L1 and LCN2 are strongly produced by tumor cells and TAM, and besides their immunomodulatory function both biomarker proteins have been linked to several tumor-promoting processes such as EMT, (lymph)angiogenesis and matrix remodeling [48–54]. Moreover, the latter process is related to MMP-9 and it has been shown that CHI3L1 induces MMP-9 production by macrophages, whereas LCN2 stabilizes this key matrix degrading protein and regulates its activity [48, 52]. Accordingly, in the current study the serum MMP-9 levels follow a similar progressive trend as the serum CHI3L1 and LCN2 levels with significantly increased levels prior to and upon metastasis in the 4T1 + RAW264.7 compared to the 4T1-only inoculated mice.

## Conclusion

Our results highlight the importance of macrophages in metastatic breakthrough of intraductally inoculated 4T1 mammary tumor cells, but also the leukemoid reactions that are associated with systemic spreading. More specifically, we show here that the signaling between the tumor cells and the macrophages in the mammary ducts results in (1) increased ductal breakthrough and metastasis with severe splenomegaly, (2) a M1 to M2 macrophage polarization and establishment of an anti-inflammatory microenvironment in support of metastasis, (3) augmented systemic levels of immune-related biomarkers CHI3L1 and LCN2 mirroring disease progression and leukemoid responses. Based on these findings, future study is warranted to explore tumor-associated macrophages and their crosstalk with tumor cells as potential immunotherapeutic target for TNBC.

## Additional files

Additional file 1: **Figure S1.** Immunohistochemistry for immune cells and vascular endothelial cells in lung metastases of 4T1 + RAW264.7 versus 4T1 intraductally inoculated mice. Immunohistochemistry for immune cell markers CD45 (pan-immune cell marker), CD163 (anti-inflammatory macrophage marker), Ly6G (neutrophil marker) and CD8a (cytotoxic T-cell marker) and the vascular endothelial cell marker CD31 was performed on paraffin sections of lung metastases from 4T1 + RAW264.7 and 4T1 inoculated mice at 5 w p.i. to identify infiltrating immune cells and vascular growth associated with 4T1 metastatic outgrowth in lungs. All scale bars = 50 μm. (TIF 22439 kb)

Additional file 2: **Figure S2.** M1−/M2-related cytokine levels in serum of 4T1 + RAW264.7, 4T1 and RAW264.7 intraductally inoculated mice. (A, B) Pro-inflammatory/M1-related (MCP-1 and MIP-2) (A) and anti-inflammatory/M2-related cytokine levels (BAFF and TGF-β1) (B) at 3 w p.i. in serum from 4T1 + RAW264.7, 4T1 and RAW264.7 inoculated mice (*n* = 5 sera for each inoculation group). (C, D) Pro-inflammatory/M1-related (MCP-1, MIP-2 and TNF-α) (C) and anti-inflammatory/M2-related cytokine levels (BAFF, G-CSF and TGF-β1) (D) at 5 w p.i. in serum from 4T1 + RAW264.7, 4T1 and RAW264.7 inoculated mice (4T1 + RAW264.7 and 4T1 inoculation group: *n* = 5 sera; RAW264.7 inoculation group: *n* = 4 sera). All data are presented as the means +/− SEM. NS: not significant, *: *P* < 0.05, ***: *P* < 0.001. (TIF 10349 kb)

Additional file 3 **Figure S3.** Immunohistochemistry of RAW264.7 inoculated mammary glands and western blot verification of M2-related/anti-inflammatory CD163 levels in 4T1 + RAW264.7 versus 4T1 primary tumors. (A) H&E histology and immunohistochemistry for the cell proliferation marker Ki67 and macrophage markers (CD45, F4/80 and CD163) were performed on paraffin sections of RAW264.7 inoculated mammary glands to verify the presence, growth and immune status of the intraductally inoculated RAW264.7 macrophages. All scale bars = 50 μm. (B) Western blot for the anti-inflammatory CD163 levels and GAPDH loading control levels in primary tumor lysates of 4T1 + RAW264.7 and 4T1 inoculated mice at 3 and 5 w p.i. (*n* = 2 at each time point and for each inoculation group), and in mammary gland lysates of RAW264.7 inoculated mice at 5 w p.i. (*n* = 1) for verification of the CD163 immunohistochemistry results. The CD163 signals were quantified relative to the GAPDH signals. Data in panel B are presented as the means +/− SEM. NS: not significant, *: *P* < 0.05. (TIF 16459 kb)

## Abbreviations

BAFF: B-cell activating factor
CAIX: Carbonic anhydrase IX
CHI3L1: Chitinase 3-like 1
DCIS: Ductal carcinoma in situ
EMT: Epithelial-to-mesenchymal transition
GAPDH: Glyceraldehyde 3-phosphate dehydrogenase
G-CSF: Granulocyte colony-stimulating factor
H&E: Hematoxylin and eosin
IFN: Interferon
IL: Interleukin
LCN2: Lipocalin 2
MCP: Monocyte chemoattractant protein
MIP: Macrophage inflammatory protein
MMP-9: Matrix metalloproteinase 9
p.i.: post-inoculation
TAM: Tumor-associated macrophages
TGF: Transforming growth factor
TNBC: Triple-negative breast cancer
TNF: Tumor necrosis factor
VEGF: Vascular endothelial growth factor
