# Supplementary figures and images for: Anti-inflammatory signaling by mammary tumor cells mediates prometastatic macrophage polarization in an innovative intraductal mouse model for triple-negative breast cancer
Source: J Exp Clin Cancer Res. 2018 Aug 15;37:191. doi: 10.1186/s13046-018-0860-x (PMC6094904; doi:10.1186/s13046-018-0860-x)

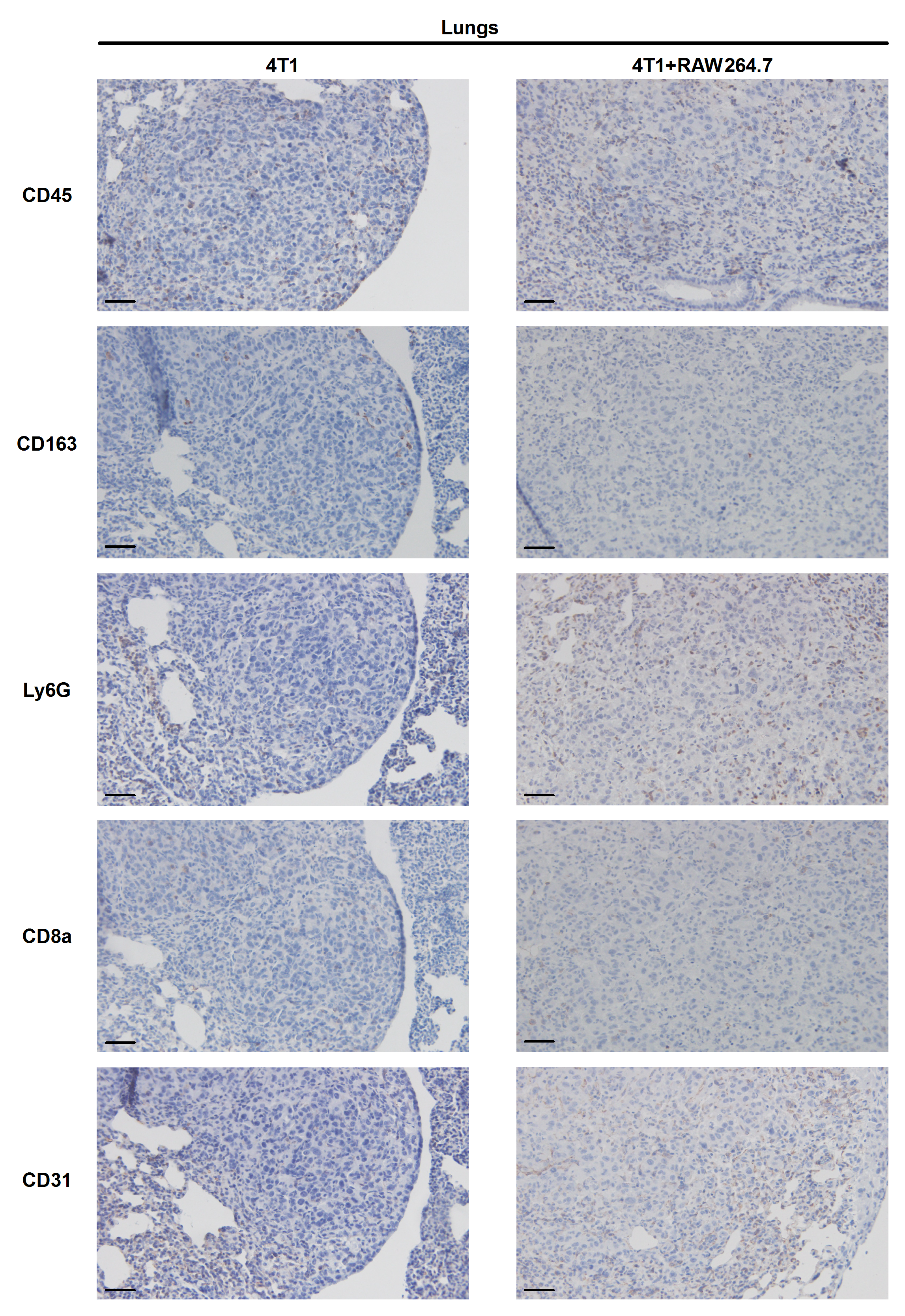

Supplement: Supplementary file 1 — Figure S1. Immunohistochemistry for immune cells and vascular endothelial cells in lung metastases of 4T1 + RAW264.7 versus 4T1 intraductally inoculated mice. Immunohistochemistry for immune cell markers CD45 (pan-immune cell marker), CD163 (anti-inflammatory macrophage marker), Ly6G (neutrophil marker) and CD8a (cytotoxic T-cell marker) and the vascular endothelial cell marker CD31 was performed on paraffin sections of lung metastases from 4T1 + RAW264.7 and 4T1 inoculated mice at 5 w p.i. to identify infiltrating immune cells and vascular growth associated with 4T1 metastatic outgrowth in lungs. All scale bars = 50 μm. (TIF 22439 kb) [file 13046_2018_860_MOESM1_ESM.tif]

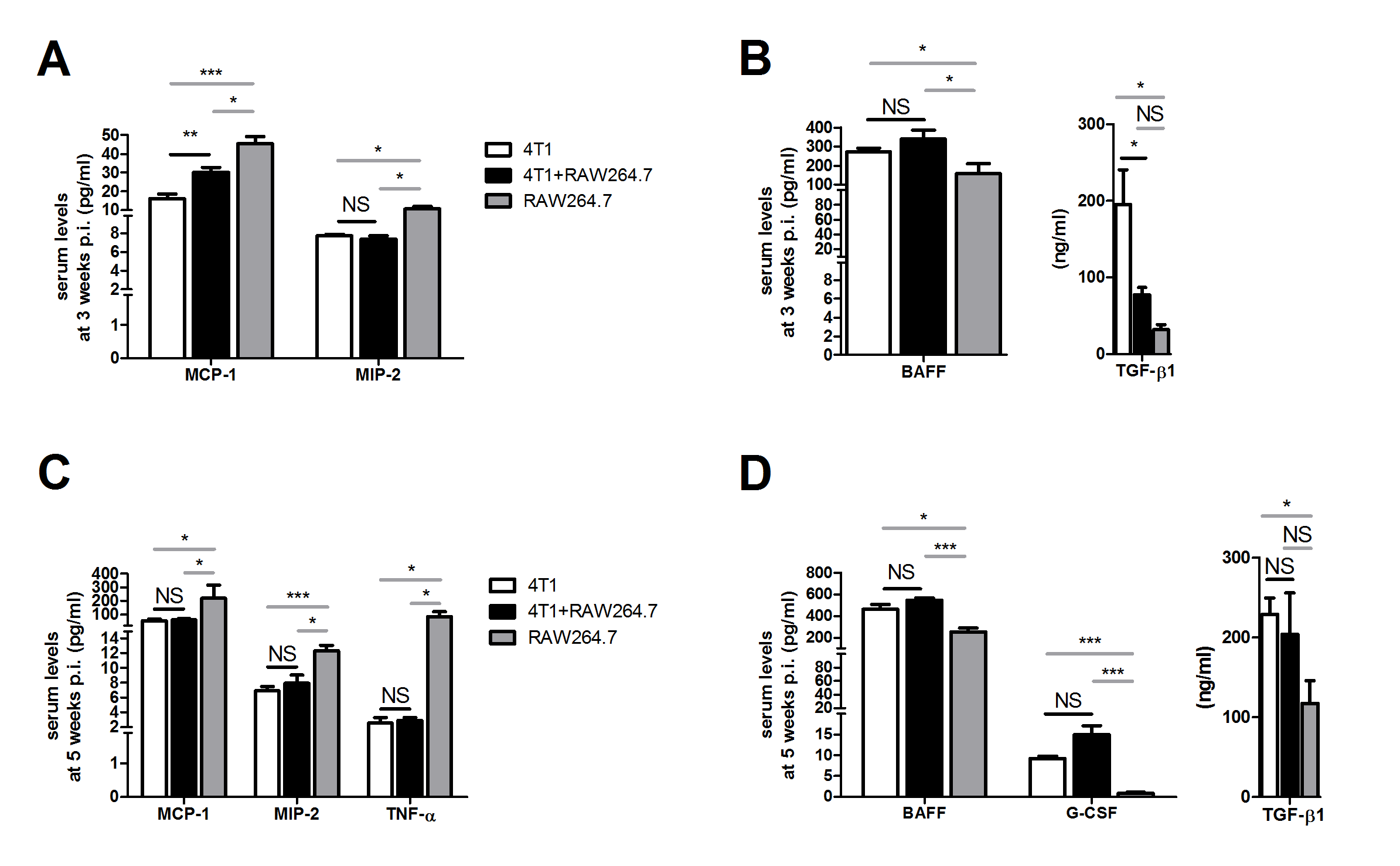

Supplement: Supplementary file 2 — Figure S2. M1−/M2-related cytokine levels in serum of 4T1 + RAW264.7, 4T1 and RAW264.7 intraductally inoculated mice. (A, B) Pro-inflammatory/M1-related (MCP-1 and MIP-2) (A) and anti-inflammatory/M2-related cytokine levels (BAFF and TGF-β1) (B) at 3 w p.i. in serum from 4T1 + RAW264.7, 4T1 and RAW264.7 inoculated mice (n = 5 sera for each inoculation group). (C, D) Pro-inflammatory/M1-related (MCP-1, MIP-2 and TNF-α) (C) and anti-inflammatory/M2-related cytokine levels (BAFF, G-CSF and TGF-β1) (D) at 5 w p.i. in serum from 4T1 + RAW264.7, 4T1 and RAW264.7 inoculated mice (4T1 + RAW264.7 and 4T1 inoculation group: n = 5 sera; RAW264.7 inoculation group: n = 4 sera). All data are presented as the means +/− SEM. NS: not significant, *: P < 0.05, ***: P < 0.001. (TIF 10349 kb) [file 13046_2018_860_MOESM2_ESM.tif]

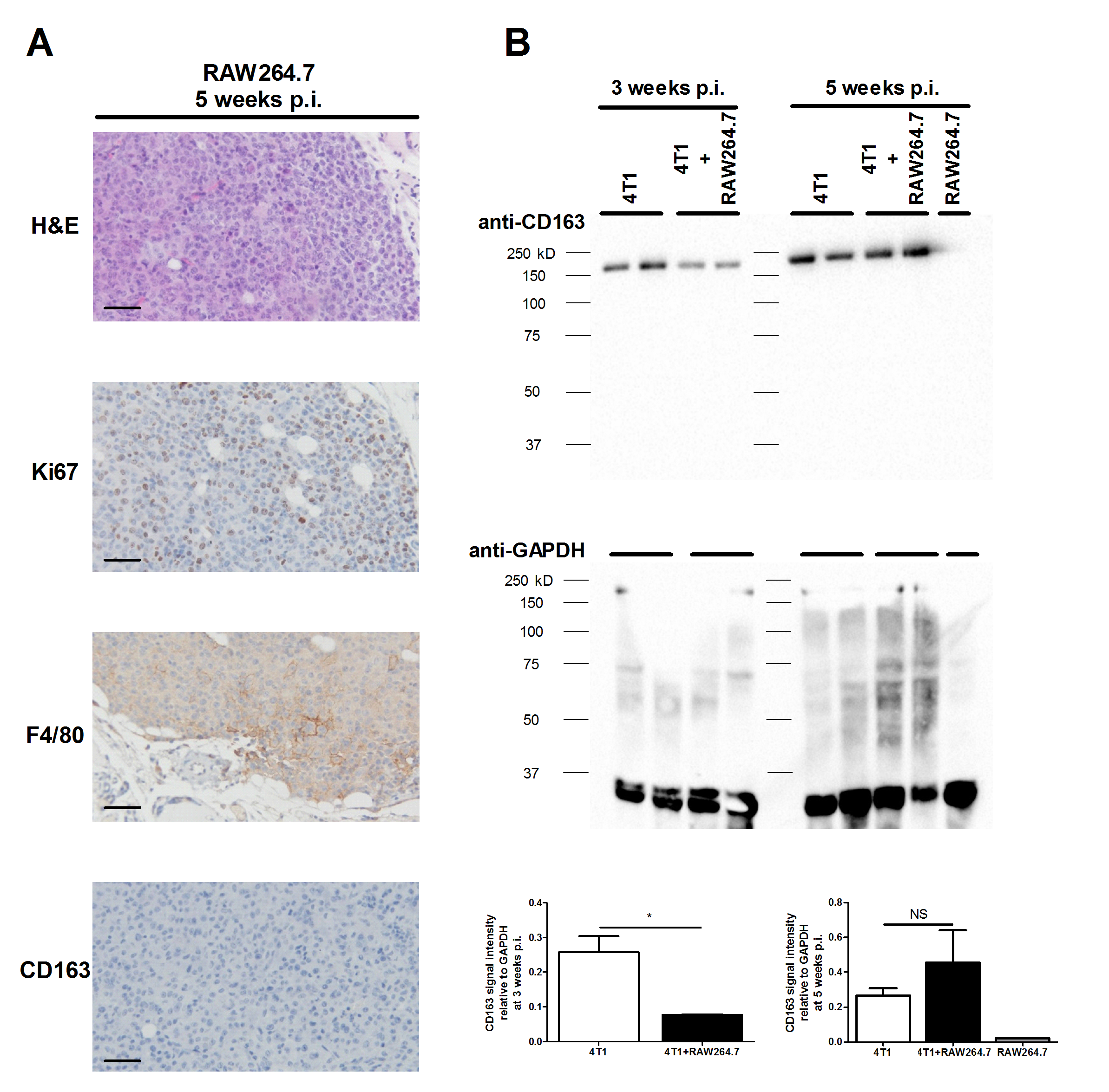

Supplement: Supplementary file 3 — Figure S3. Immunohistochemistry of RAW264.7 inoculated mammary glands and western blot verification of M2-related/anti-inflammatory CD163 levels in 4T1 + RAW264.7 versus 4T1 primary tumors. (A) H&E histology and immunohistochemistry for the cell proliferation marker Ki67 and macrophage markers (CD45, F4/80 and CD163) were performed on paraffin sections of RAW264.7 inoculated mammary glands to verify the presence, growth and immune status of the intraductally inoculated RAW264.7 macrophages. All scale bars = 50 μm. (B) Western blot for the anti-inflammatory CD163 levels and GAPDH loading control levels in primary tumor lysates of 4T1 + RAW264.7 and 4T1 inoculated mice at 3 and 5 w p.i. (n = 2 at each time point and for each inoculation group), and in mammary gland lysates of RAW264.7 inoculated mice at 5 w p.i. (n = 1) for verification of the CD163 immunohistochemistry results. The CD163 signals were quantified relative to the GAPDH signals. Data in panel B are presented as the means +/− SEM. NS: not significant, *: P < 0.05. (TIF 16459 kb) [file 13046_2018_860_MOESM3_ESM.tif]
